# Polar Codes for Decomposed Multi-Input Multi-Output Gaussian Broadcast Channels †

**DOI:** 10.3390/e28070798

**Published:** 2026-07-14

**Authors:** Muhammed Yusuf Şener, Gerhard Kramer, Shlomo Shamai (Shitz), Ronald Böhnke, Wen Xu

**Affiliations:** 1 Institute for Communications Engineering, School of Computation, Information and Technology, Technical University of Munich, 80333 Munich, Germany; gerhard.kramer@tum.de; 2 Huawei Technologies Duesseldorf GmbH, 80992 Munich, Germanywen.xu@ieee.org (W.X.); 3 Department of Electrical and Computer Engineering, Technion—Israel Institute of Technology, Haifa 3200003, Israel

**Keywords:** broadcast channel, capacity, dirty paper coding, multi-input multi-output

## Abstract

Dirty paper coding (DPC) is applied to multi-input multi-output (MIMO) broadcast channels with additive Gaussian noise and one message per receiver. The method decomposes each receiver MIMO channel into parallel scalar channels and applies modulo operators, amplitude-shift keying (ASK), and probabilistic shaping. The achievable rate tuples include all points inside the capacity region by choosing truncated Gaussian shaping, large ASK alphabets, and large modulo intervals. Simulations with short polar codes show significant rate and power gains from DPC compared to linear precoding, while maintaining similar encoding and decoding complexities.

## 1. Introduction

This paper is dedicated to Professor H. Vincent Poor on the occasion of his 75th birthday. The authors would like to express their appreciation for his outstanding contributions to engineering research and his unwavering commitment to the information theory and communications communities.

The paper studies the downlink of wireless systems with multiple antennas, i.e., the multi-input multi-output (MIMO) setting. We compare linear precoding (LP) and dirty paper coding (DPC) when using polar codes. Professor Poor authored numerous influential papers on LP and DPC, covering topics such as multiuser detection [1,2,3], code-division multiple access (CDMA) [4,5,6], orthogonal and non-orthogonal multiple access (OMA and NOMA) [7,8,9,10], rate-splitting multiple access (RSMA) [11,12,13], and non-linear precoding [14].

### 1.1. Linear Precoding

LP is a superposition coding method that linearly combines encoded signals. It involves modulating beams, or vectors, and has two basic variants. The first variant, which we focus on, selects one beam per message and treats interference as noise. This approach is referred to as space division multiple access (SDMA) in [11], and we will refer to it simply as LP. The second variant avoids interference by using orthogonal beams, such as time- or frequency-division multiplexing, and is often called OMA. It is worth noting that SDMA, OMA, and other variants of LP can achieve higher rates by utilizing multiple transmission modes with optimized resource allocation across time, frequency, and power [15]. However, adding modes introduces latency. We also note that using different modes can be represented by a “mode-sharing” or “time-sharing” random variable [16] (p. 278). Such a variable is generally preferable to a convex hull operator to compute achievable rates, because a convex hull does not capture all mode-sharing possibilities. An example is supplied in [16] (p. 288), where the convex hull gives a suboptimal rate region for a compound multiple-access channel (MAC).

SDMA and OMA are commonly used in practice, but they yield suboptimal results. An LP variation that achieves the capacity of broadcast channels (BCs) with single-antenna transceivers utilizes successive interference cancellation (SIC), as discussed by Cover [17] (Section VII), Bergmans [18,19], Gallager [20], and others [21,22]. In this approach, each UE first decodes a set of signals intended for other UEs, then cancels the interference from those signals before decoding its own signal. This method is commonly referred to as NOMA. However, SIC increases the complexity and latency for the UEs.

A variation of NOMA involves splitting each message into multiple parts, encoding them separately, and decoding them using SIC. This approach effectively increases the number of messages being transmitted, similar to using multiple transmission modes. Message splitting was introduced for interference channels [23] and was soon applied to broadcast channels [24] (Section III). The method is sometimes referred to as RSMA [11,12,25,26]. RSMA increases the complexity and latency of base stations (BSs) and user equipments (UEs), but it can offer performance advantages when channel state information at the transmitter (CSIT) is imperfect [27,28].

The most general approach combines superposition coding with DPC, and we refer to this method as Marton coding [29]; see also [16] (p. 391) and [28] (Section II.C). Marton coding can be implemented by targeting specific corner points of the rate region using DPC, and then applying time-sharing. We remark that time-sharing latency can be significantly reduced by utilizing fine time-shifts and alternating DPC [30].

### 1.2. Dirty Paper Coding

DPC arises in the context of channels with non-causal state information at the transmitter, for which the capacity was characterized by Gelfand and Pinsker [31]. Costa applied the theory to additive white Gaussian noise (AWGN) channels with additive Gaussian interference known at the transmitter. Remarkably, the capacity is the same as without interference [32], a result similar to Wyner–Ziv coding for Gaussian sources and mean square error distortion [33]. The result was later extended to general interference by using high-dimensional lattices [34,35,36].

DPC achieves all points in the capacity region of a linear, MIMO, AWGN BC with independent messages, each destined for a different receiver [37,38,39,40,41,42,43]. The idea is to encode the messages successively, treating the signals of previously encoded messages as interference. One thereby achieves higher rates than LP. However, DPC is rarely used in practice because it is considered too complex [26] (Section III.A). We show that DPC can be implemented with a similar level of complexity to coded LP, albeit with higher latency due to successive encoding.

A useful insight for understanding DPC is that successive encoding is the dual of successive decoding at the receiver of an appropriate (dual) MAC [41]. Remarkably, Gaussian signaling, linear minimum mean-square error (LMMSE) estimation, and convex optimization yield the best MAC rates, and the best MAC beamforming vectors can be transformed into the best BC precoding vectors. A refined approach decomposes the BC into scalar channels and shows that the best BC precoding and MAC beamforming vectors are co-linear [44,45].

### 1.3. Code Constructions

Explicit DPC constructions are described in [46,47,48,49]. These papers use low-density parity-check (LDPC) or turbo codes for reliability and trellis codes for shaping. The schemes require long block lengths, and the complexity is high. Similarly, the superposition coding scheme in [50] employs long LDPC and trellis codes. Lattice constructions with integer-forcing were proposed in [51,52,53,54]; these perform well, but typically do not achieve capacity. Non-asymptotic analyses of random coding are described in [55,56,57,58,59,60,61,62,63].

Polar codes [64] are an attractive alternative because they permit practical nested coding for reliability, quantization, and shaping. The paper [65] analyzes binary DPC, the paper [66] shows how to perform shaping, the paper [67] introduces polar-coded modulation, and the thesis [68] designs polar lattices for DPC; see also [30,69,70,71,72,73,74,75,76,77,78,79,80,81,82,83,84,85,86,87]. We also use polar codes, but with scalar modulo operations, dithering, and probabilistic shaping [60,62]. A modulo operator reduces the transmitter dynamic range, and dithering decouples the constellation and interference statistics, which provides useful independencies.

### 1.4. Organization and Contributions

This paper is organized as follows. Section 2 reviews notation, and results on covariance matrices and symmetric unimodal functions. Section 3 describes the vector BC and shows that noise whitening and the singular value decomposition (SVD) give parallel scalar channels with known interference. One may thus apply scalar DPC to achieve all rate tuples in the capacity region [88]. The main technical contributions are provided in Section 4 and Section 5, where we generalize Lemmas 2 and Theorem 1 from [62] to include non-Gaussian noise. The extensions are used to prove that the DPC scheme of [62] achieves all points in the capacity region of MIMO BCs. Section 6 computes the capacity region for two example BCs, including a BC with orthogonal frequency-division multiplexing (OFDM). Section 7 designs short polar codes for the OFDM BC, and provides frame error rate (FER) curves based on numerical simulations. This section also compares the complexities of LP and DPC when using polar codes. Section 8 concludes the paper.

## 2. Preliminaries

### 2.1. Notation

Column vectors are denoted as $\underline{x}={\left[x_{1},\ldots ,x_{n}\right]}^{T}$, where ${\underline{x}}^{T}$ is the transpose of $\underline{x}$. The complex conjugate transpose of $\underline{x}$ is written as ${\underline{x}}^{†}$. Bold letters such as Q denote matrices; $Q^{†}$ is the complex-conjugate transpose of Q; trace(Q) is the trace of Q; det(Q) is the determinant of Q; and I is an identity matrix. We write $j=\sqrt{-1}$, ${\left[x\right]}^{+}=\max\left(0,x\right)$, and

(1) $$\begin{array}{c} x\;\mathrm{mod}\;A=x-k^{\prime }A \end{array}$$

where $k^{\prime }$ is the integer so that $x-k^{\prime }A$ lies in [−A/2,A/2).

Upper- and lowercase letters denote random variables (RVs) and their realizations; e.g., $\underline{X}$ is a random vector and $\underline{x}$ is its realization. $p_{X}$ denotes the probability density function of the random variable *X*. We remove subscripts if the argument is a lowercase version of the RV, e.g., $p\left(x\right)=p_{X}\left(x\right)$. Define the expectation and entropy with respect to a density q(.) as

(2) $$\begin{array}{cc} E_{q}\left[f\left(X\right)\right] & :=\int_{R}q\left(x\right)\;f\left(x\right)\;dx \end{array}$$

(3) $$\begin{array}{cc} h_{q}\left(X\right) & :=E_{q}\left[-\log q\left(X\right)\right]. \end{array}$$

We write E[X] and h(X) if the density is clear from the context. I(X;Y) denotes the mutual information of *X* and *Y*. Q(·) denotes the *Q*-function.

### 2.2. Decomposition of a Covariance Matrix

Let $\underline{m}=E\left[\underline{X}\right]$ and $K=E[\left(\underline{X}-\underline{m}\right){\left(\underline{X}-\underline{m}\right)}^{†}]$ be the mean and covariance matrix of $\underline{X}$. The matrix K is positive semi-definite and has the eigenvalue decomposition

(4) $$\begin{array}{c} K=U\Lambda U^{†} \end{array}$$

where U is unitary and Λ is diagonal with non-negative entries. Suppose there are $n_{+}$ positive eigenvalues. Let $\bar{\Lambda }$ be the $n_{+}\times n_{+}$ matrix with these eigenvalues, and let $\bar{U}$ be the $n\times n_{+}$ matrix with the appropriate columns of U. Then a Cholesky decomposition is

(5) $$\begin{array}{c} K=PP^{†}\;\mathrm{where}\;P=\bar{U}{\bar{\Lambda }}^{1/2}{\bar{V}}^{†} \end{array}$$

for any $n_{+}\times n_{+}$ unitary matrix $\bar{V}$. Observe that P is an $n_{t}\times n_{+}$ matrix with the left inverse

(6) $$\begin{array}{c} P^{+}=\bar{V}{\bar{\Lambda }}^{-1/2}{\bar{U}}^{†}. \end{array}$$

For example, if $n_{+}=n$ and $\bar{V}=U$ then $K=P^{2}$. In this case, one usually writes $P=K^{1/2}$ and $P^{+}=P^{-1}=K^{-1/2}$.

### 2.3. Symmetric and Unimodal Functions

We study symmetric and unimodal probability density functions (p.d.f.s). A real-valued function f(.) is said to be symmetric if f(x)=f(−x) for all x∈R. A symmetric p.d.f p(.) is called unimodal if p(x) is non-increasing for x≥0, and hence non-decreasing for x≤0. The following lemmas are known; we provide proofs in Appendix A.

**Lemma** **1.** *The convolution f∗g(.) of two symmetric functions f(.) and g(.) is symmetric.*

**Lemma** **2.** *The convolution p∗q(.) of two symmetric unimodal p.d.f.s p(.) and q(.) is symmetric unimodal.*

**Lemma** **3.** *Consider a symmetric and unimodal p.d.f. f(.). We have*

(7) $$\begin{array}{c} \left|\int_{-\frac{A}{2}}^{\frac{A}{2}}f\left(y\right)dy-\sum_{k=-\lfloor M/2\rfloor }^{\lceil M/2\rceil -1}\kappa f\left(x+k\kappa \right)\right|\leq \kappa f\left(0\right) \end{array}$$

*for x satisfying 0≤x<κ/2. Similarly, if f(.) has finite area then for any x we have*

(8) $$\begin{array}{c} \left|\int_{R}f\left(y\right)dy-\sum_{k\in Z}\kappa f\left(x+k\kappa \right)\right|\leq \kappa f\left(0\right). \end{array}$$

### 2.4. Scalar DPC Scheme

**Figure 1:**
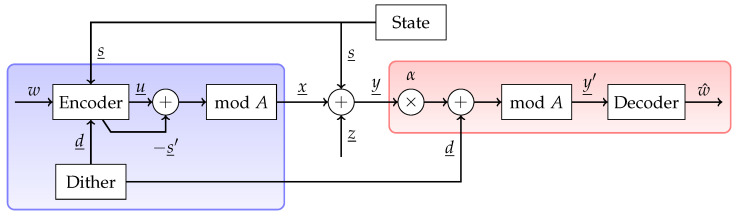
DPC with probabilistic shaping, a modulo operator, and dithering. The blue and red shaded boxes show the transmitter and receiver, respectively.

We review the scalar DPC scheme from [62] that is depicted in Figure 1. The structure is a scalar version of a multi-dimensional nested lattice scheme [34] but adds probabilistic shaping to remove interference and improve power efficiency. The dither decouples the shaping and interference statistics, which simplifies analysis and signal processing at the decoder. The dither also enables secrecy and provides robustness [36].

Consider the real-alphabet channel

(9) $$\begin{array}{c} Y=X+S+Z \end{array}$$

where *X* satisfies the power constraint $E\left[X^{2}\right]\leq P_{X}$, *S* is interference known at the transmitter, and *Z* (not necessarily Gaussian) is independent of (X,S) and has variance $P_{Z}$. Figure 1 shows the channel inputs and outputs as vectors $\underline{x}$ and $\underline{y}$, respectively. The noise $\underline{z}$, state $\underline{s}$, and dither $\underline{d}$ are also shown as vectors. We explain the scheme in terms of scalar random variables that represent the vectors’ entries. For example, we consider a per-entry dither *D* that is continuously uniform over the interval [−A/2,A/2).

Consider a shaping density q(x) and the *M*-ary amplitude shift keying (*M*-ASK) constellation

(10) $$\begin{array}{c} U={\left\{-\frac{A}{2}+\left(k+1/2\right)\kappa \right\}}_{k=0}^{M-1},\;\kappa =\frac{A}{M} \end{array}$$

where κ is the ASK-spacing. The encoder computes (see [62] (Equation (1)))

(11) $$\begin{array}{cc} S^{\prime } & =\left(\alpha S+D\right)\;\mathrm{mod}\;A \end{array}$$

and selects a symbol *U* by using the message *W* and the discrete shaping distribution

(12) $$\begin{array}{c} P\left(u|s^{\prime }\right)=\frac{q\left(\left(u-s^{\prime }\right)\;\mathrm{mod}\;A\right)}{\sum_{v\in U}q\left((v-s^{\prime })\;\mathrm{mod}\;A\right)},\;u\in U. \end{array}$$

The transmitted signal is (see [62] (Equation (2)))

(13) $$\begin{array}{cc} X & =(U-S^{\prime })\;\mathrm{mod}\;A \end{array}$$

where $\alpha =P_{X}/\left(P_{X}+P_{Z}\right)$. The received signal is (see [62] (Equations (3) and (4)))

(14) $$\begin{array}{cc} Y^{\prime } & =\left(\alpha (X+S+Z)+D\right)\;\mathrm{mod}\;A\\ \; & =\left(\alpha X+S^{\prime }+\alpha Z\right)\;\mathrm{mod}\;A\\ \; & =(U+Z^{\prime })\;\mathrm{mod}\;A \end{array}$$

where the interference *S* has been removed, and the effective noise is

(15) $$\begin{array}{c} Z^{\prime }=\left(\alpha -1\right)X+\alpha Z \end{array}$$

with variance $\sigma_{Z^{\prime }}^{2}=\alpha P_{Z}$. The receiver puts out the message estimate $\hat{W}$.

The shaping (12) induces the input density (see [62] (Equations (8) and (10)))

(16) $$\begin{array}{c} p(x)=q(x)/d(x),\;x\in [-A/2,A/2) \end{array}$$

where the denominator is

(17) $$\begin{array}{c} d\left(x\right)=\sum_{k=0}^{M-1}\kappa \;q\left(\left(x+k\kappa \right)\;\mathrm{mod}\;A\right). \end{array}$$

We study truncated Gaussian shaping with parameters *A* and $\sigma_{q}$, namely (see [62] (Equation (12)))

(18) $$\begin{array}{c} q\left(x\right)=\frac{e^{-x^{2}/\left(2\sigma_{q}^{2}\right)}}{c\sqrt{2\pi \sigma_{q}^{2}}}\;,\;x\in \left[-A/2,A/2\right) \end{array}$$

where $c=1-2Q(A/\left(2\sigma_{q}\right))$. We have $E_{q}\left[X\right]=0$, and compute the variance and entropy (see [62] (Equations (16) and (17)))

(19) $$\begin{array}{cc} E_{q}\left[X^{2}\right] & =\sigma_{q}^{2}\left[1-\frac{A\;e^{-A^{2}/\left(8\sigma_{q}^{2}\right)}}{c\sqrt{2\pi \sigma_{q}^{2}}}\right] \end{array}$$

(20) $$\begin{array}{cc} h_{q}\left(X\right) & =\frac{1}{2}\log\left(2\pi e\;\sigma_{q}^{2}c^{2}\right)-\frac{1}{2}\left(1-\frac{E_{q}\left[X^{2}\right]}{\sigma_{q}^{2}}\right)\log e. \end{array}$$

## 3. Gaussian Vector Broadcast Channels

Consider the complex-alphabet BC

(21) $$\begin{array}{c} {\underline{Y}}_{k}=H_{k}\underline{X}+{\underline{Z}}_{k},\;k=1,\ldots ,K. \end{array}$$

where $\underline{X}$ has dimension $n_{t}$, ${\underline{Y}}_{k}$ and ${\underline{Z}}_{k}$ have dimension $n_{k}$, $H_{k}$ is an $n_{k}\times n_{t}$ complex matrix, and ${\underline{Z}}_{k}$ is a circularly symmetric, complex, Gaussian (CSCG) noise vector with invertible covariance matrix $Q_{k}$. We require $\underline{X}$ to satisfy the power constraint $E[∥\underline{X}{∥}^{2}]\leq P_{X}$.

Consider one message per UE, where the message of UE *k* has rate $R_{k}$. Let $K_{k}$ be an $n_{t}\times n_{t}$ covariance matrix associated with message *k*. The following rate tuples $\left(R_{1},\ldots ,R_{K}\right)$ are achieved by considering the UE ordering 1,…,K, and removing the interference of messages *l* with l<k:

(22) $$\begin{array}{c} R_{k}\leq \log\det\left(I+H_{k}K_{k}H_{k}^{†}{\left(Q_{k}+\sum_{l>k}H_{k}K_{l}H_{k}^{†}\right)}^{-1}\right) \end{array}$$

subject to $\sum_{k=1}^{K}\mathrm{trace}\left(K_{k}\right)\leq P_{X}$. The capacity region is the union of all such rate tuples, taken over all $K_{1},\ldots ,K_{K}$ and UE orderings; see [29,42].

### 3.1. Capacity-Achieving Scheme

The BS sends $\underline{X}=\sum_{k=1}^{K}{\underline{X}}_{k}$ where the ${\underline{X}}_{k}$ are statistically independent [41] and ${\underline{X}}_{k}$ has the covariance matrix $K_{k}$. The optimal ${\underline{X}}_{k}$ are CSCG. Using (5), we have

(23) $$\begin{array}{c} K_{k}=P_{k}P_{k}^{†}\;\mathrm{where}\;P_{k}={\bar{U}}_{k}{\bar{\Lambda }}_{k}^{1/2}{\bar{V}}_{k}^{†} \end{array}$$

where ${\bar{U}}_{k}$, ${\bar{\Lambda }}_{k}$, and ${\bar{V}}_{k}$ have dimensions $n_{t}\times n_{k+}$, $n_{k+}\times n_{k+}$, and $n_{k+}\times n_{k+}$, respectively. Of course, we have $n_{k+}\leq n_{k}$ for all *k*.

Consider the ordering 1,…,K. UE *k* sees

(24) $${\underline{Y}}_{k}\;=H_{k}{\underline{X}}_{k}+\underset{:={\underline{S}}_{k}}{\underset{︸}{\left(\sum_{l<k}H_{k}{\underline{X}}_{l}\right)}}+\underset{:={\underline{\overset{ˇ}{Z}}}_{k}}{\underset{︸}{\left(\sum_{l>k}H_{k}{\underline{X}}_{l}\right)+{\underline{Z}}_{k}}}$$

and treats ${\underline{S}}_{k}$ as interference and ${\underline{\overset{ˇ}{Z}}}_{k}$ as noise. Let ${\overset{ˇ}{Q}}_{k}$ be the $n_{k}\times n_{k}$ covariance matrix of ${\underline{\overset{ˇ}{Z}}}_{k}$. We use the SVD to write

(25) $$\begin{array}{c} {\overset{ˇ}{Q}}_{k}^{-1/2}H_{k}{\bar{U}}_{k}{\bar{\Lambda }}_{k}^{1/2}=U_{k}\Sigma_{k}V_{k}^{†} \end{array}$$

where $U_{k}$ and $V_{k}$ are unitary and $\Sigma_{k}$ is an $n_{k}\times n_{k+}$ diagonal matrix with the singular values of ${\overset{ˇ}{Q}}_{k}^{-1/2}H_{k}{\bar{U}}_{k}{\bar{\Lambda }}_{k}^{1/2}$. Now choose ${\bar{V}}_{k}=V_{k}^{†}$ in (23). UE *k* left-multiplies ${\underline{Y}}_{k}$ with the $n_{k}\times n_{k}$ noise-whitening filter

(26) $$\begin{array}{c} F_{k}=U_{k}^{†}{\overset{ˇ}{Q}}_{k}^{-1/2} \end{array}$$

to obtain the parallel channel

(27) $$\begin{array}{c} {\underline{\tilde{Y}}}_{k}=\Sigma_{k}{\underline{\tilde{X}}}_{k}+{\underline{\tilde{S}}}_{k}+{\underline{\tilde{Z}}}_{k} \end{array}$$

where ${\underline{\tilde{S}}}_{k}$ is known at the BS and

(28) $$\begin{array}{c} {\underline{\tilde{X}}}_{k}=P_{k}^{+}{\underline{X}}_{k},\;{\underline{\tilde{S}}}_{k}=F_{k}{\underline{S}}_{k},\;{\underline{\tilde{Z}}}_{k}=F_{k}{\underline{\overset{ˇ}{Z}}}_{k}. \end{array}$$

Note that only the first $n_{k+}$ entries of ${\underline{\tilde{Y}}}_{k}$ are useful channel outputs, i.e., we have $n_{\mathrm{ch}}:=\sum_{k=1}^{K}n_{k+}$ useful sub-channels.

The covariance matrices of ${\underline{\tilde{X}}}_{k}$ and ${\underline{\tilde{Z}}}_{k}$ are identity matrices, i.e., the entries ${\tilde{X}}_{k,i}$, $i=1,\ldots ,n_{k+}$, and ${\tilde{Z}}_{k,i}$, $i=1,\ldots ,n_{k}$, are independent and identically distributed (i.i.d.) CSCG with unit variance. The power constraint is $\sum_{k=1}^{K}P_{k}\leq P_{X}$ where $P_{k}=\mathrm{trace}K_{k}$. The BS can use $P_{k}$ as an $n_{t}\times n_{k+}$ precoding matrix to compute ${\underline{X}}_{k}=P_{k}{\underline{\tilde{X}}}_{k}$.

### 3.2. Parallel DPC with Scalar Modulo Operations

The ${\underline{\tilde{X}}}_{k}$ are independent since the ${\underline{X}}_{k}$ are independent. Moreover, the decomposition (27) specifies $n_{\mathrm{ch}}$ sub-channels where the *i*th entry of ${\underline{\tilde{Y}}}_{k}$ is

(29) $$\begin{array}{c} {\tilde{Y}}_{k,i}=\sigma_{k,i}{\tilde{X}}_{k,i}+{\tilde{S}}_{k,i}+{\tilde{Z}}_{k,i} \end{array}$$

for k=1,…,K and $i=1,\ldots ,n_{k+}$. The parameter $\sigma_{k,i}$ is a singular value, ${\tilde{S}}_{k,i}$ is known at the BS, and ${\tilde{Z}}_{k,i}$ is CSCG and independent of ${\tilde{X}}_{k,i},{\tilde{S}}_{k,i}$. Thus, one achieves capacity if one achieves the capacity $\log(1+\sigma_{k,i}^{2})$ for each scalar channel (29) in (27), and for the covariance matrices $K_{1},\ldots ,K_{K}$ of all required rate tuples.

One may write ${\underline{Z}}_{k}=Q_{k}^{1/2}{\underline{W}}_{k}$ where the entries $W_{k,i}$ of ${\underline{W}}_{k}$ are i.i.d. CSCG with unit variance. Thus, using (24) and (28), we have

(30) $$\begin{array}{c} {\underline{\tilde{Z}}}_{k}=F_{k}\left[\left(\sum_{l>k}H_{k}P_{l}{\underline{\tilde{X}}}_{l}\right)+Q_{k}^{1/2}{\underline{W}}_{k}\right]. \end{array}$$

Alternatively, there are constants $a_{k,i,l,h}$ and $b_{k,i,h}$ for which

(31) $$\begin{array}{c} {\tilde{Z}}_{k,i}=\left(\sum_{l>k}\sum_{h=1}^{n_{l+}}a_{k,i,l,h}{\tilde{X}}_{l,h}\right)+\sum_{h=1}^{n_{k}}b_{k,i,h}W_{k,h}. \end{array}$$

We apply the DPC scheme of Section 2.4 to the real and imaginary parts of each ${\tilde{X}}_{k,i}$, i.e., we use $M^{2}$-ary quadrature amplitude modulation ($M^{2}$-QAM) for each sub-channel (29). We similarly treat the real and imaginary parts of each ${\tilde{Y}}_{k,i}$ as independent with the same real channel gain $\sigma_{k,i}$ and noise variance 1/2. We thus focus on the real part and abuse notation by using the same symbols as for the complex alphabet channels; i.e., all random variables are now real-valued. Also, the ${\tilde{X}}_{k,i}$, ${\tilde{S}}_{k,i}$, and ${\tilde{Z}}_{k,i}$ are no longer Gaussian, with the exception of the ${\tilde{Z}}_{K,i}$.

More precisely, consider the real part of sub-channel k,i and define

(32) $$\begin{array}{c} Y:=\frac{{\tilde{Y}}_{k,i}}{\sigma_{k,i}}=X+S+Z \end{array}$$

where

(33) $$\begin{array}{c} X:={\tilde{X}}_{k,i},\;S:=\frac{{\tilde{S}}_{k,i}}{\sigma_{k,i}},\;Z:=\frac{{\tilde{Z}}_{k,i}}{\sigma_{k,i}}. \end{array}$$

Now code as in Section 2.4, where we identify $D:=D_{k,i}$ and the parameters

(34) $$\begin{array}{c} M:=M_{k,i},\;A:=A_{k,i},\;\kappa :=\kappa_{k,i},\;\alpha :=\alpha_{k,i}. \end{array}$$

Note that *Z* is not necessarily Gaussian. We choose the $D_{k,i}$ across the sub-channels as independent, which makes the ${\tilde{X}}_{k,i}$ independent for all k,i; see Appendix B. Thus, all RVs on the right-hand side of (31) are independent and the density of ${\tilde{Z}}_{k,i}$ is the convolution of the densities of the $a_{k,i,l,h}{\tilde{X}}_{l,h}$ and $b_{k,i,h}W_{k,h}$ (more generally, if a ${\tilde{X}}_{l,h}$ does not have a density, the density of ${\tilde{Z}}_{k,i}$ is the derivative of the convolution of the distribution functions of the $a_{k,i,l,h}{\tilde{X}}_{l,h}$ and $b_{k,i,h}W_{k,h}$).

We prove the following results in Section 4 and Section 5. Theorem 1 specifies achievable rates for finite $\underline{A}$ that can thus be useful under peak power constraints.

**Lemma** **4** (cf. [62] Lemma 2)**.** *Consider fixed $A_{k,i}$. In the limit of large $M_{k,i}$, we have $p_{k,i}\left(x\right)=q_{k,i}\left(x\right)$ for all x.*

**Theorem** **1** (cf. [62] Theorem 1)**.** *Consider the DPC scheme in Section 2.4 applied to all $n_{\mathrm{ch}}$ sub-channels. For each sub-channel, consider the variables (33) and (34) and the shaping density (18). Given A, the parameter $\sigma_{q}^{2}$ is chosen so that (19) takes on the value $E_{q}\left[X^{2}\right]=1/2$. Let*

$$\begin{array}{c} \underline{R}=\left(R_{1,1},\ldots ,R_{K,n_{K+}}\right) \end{array}$$

*be an $n_{\mathrm{ch}}$-tuple of achievable rates for the specified parameters*

$$\begin{array}{c} \underline{A}=\left(A_{1,1},\ldots ,A_{K,n_{K+}}\right)\;\mathrm{and}\;\underline{M}=\left(M_{1,1},\ldots ,M_{K,n_{K+}}\right). \end{array}$$

*Given $\underline{A}$, there is a sequence of achievable rates $\underline{R}$ satisfying*

(35) $$\begin{array}{c} \lim_{\underline{M}\to \underline{\infty }}R_{k,i}\geq \frac{1}{2}\log\left(2\sigma_{q}^{2}c^{2}\left(1+\sigma_{k,i}^{2}\right)\right)-\frac{1}{2}\left(1-\frac{1}{2\sigma_{q}^{2}}\right)\log e \end{array}$$

*for all k,i. Moreover, for each sub-channel, we have c→1 and $\sigma_{q}^{2}\to 1/2$ as $\underline{A}\to \underline{\infty }$, yielding*

(36) $$\begin{array}{c} \lim_{\underline{A}\to \underline{\infty }}\;\lim_{\underline{M}\to \underline{\infty }}R_{k,i}=\frac{1}{2}\log\left(1+\sigma_{k,i}^{2}\right)\;\mathrm{for}\;\mathrm{all}\;k,i. \end{array}$$

## 4. Proof of Lemma 4

**Figure 2:**
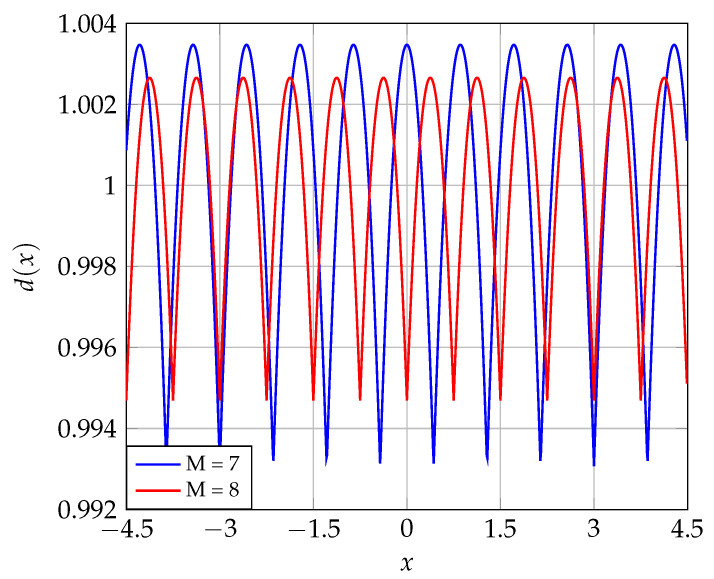
d(x) for the shaping density q(x) in (18) with A=6 and $\sigma_{q}=1.8$, and for M=7 (κ≈0.857), and M=8 (κ=0.75). Observe that d(x) is periodic with period κ and d(x)≈1.

Consider d(x) in (17), and recall that $x\;\mathrm{mod}\;\kappa =x-k^{\prime }\kappa$ for a unique integer $k^{\prime }$. We have

(37) $$\begin{array}{cc} d(x) & \overset{\left(a\right)}{=}\sum_{k=0}^{M-1}\kappa \;q\left(\left(x\;\mathrm{mod}\;\kappa +k\kappa \right)\;\mathrm{mod}\;A\right)=d\left(x\;\mathrm{mod}\;\kappa \right) \end{array}$$

where step (a) follows because the modulo operator permits summing over any *M* successive integers. Thus, d(.) is periodic with period κ. For example, Figure 2 shows d(x) for A=6, M=7,8, and the truncated Gaussian q(x) in [62] (Equation (11))with $\sigma_{q}=1.8$. Note that d(.) satisfies (37) for general q(.) and not just a truncated Gaussian q(x).

Suppose q(x) is symmetric, which means that d(x) and p(x)=q(x)/d(x) are symmetric. For x∈[0,κ/2), we have

(38) $$\begin{array}{cc} d(x) & =\sum_{k=-\lfloor M/2\rfloor }^{k=\lceil M/2\rceil -1}\kappa \;q\left(x+k\kappa \right). \end{array}$$

Now suppose q(.) is symmetric and unimodal. Applying (7) in Lemma 3, we have $d_{\min}\leq d\left(x\right)\leq d_{\max}$ for all *x*, where

(39) $$\begin{array}{c} d_{\min}=1-\kappa q\left(0\right),\;d_{\max}=1+\kappa q\left(0\right). \end{array}$$

**Figure 3:**
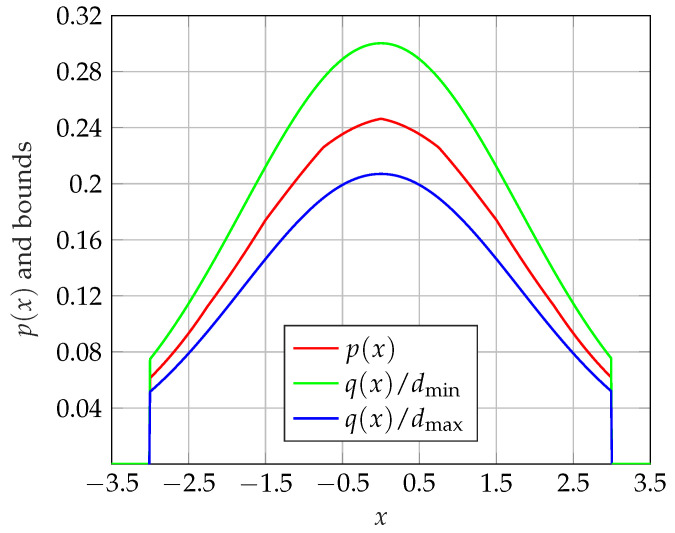
Curves of (40) for A=6, M=8, and truncated Gaussian shaping with $\sigma_{q}=1.8$.

We thus have $d_{\min},d_{\max}\to 1$ for M→∞ and

(40) $$\begin{array}{c} \frac{q(x)}{d_{\max}}\leq p\left(x\right)\leq \frac{q(x)}{d_{\min}} \end{array}$$

where we assume *M* is sufficiently large so $d_{\min}>0$. Figure 3 illustrates the bounds (40) for A=6, M=8, and the truncated Gaussian q(.) in [62] (Equation (12))with $\sigma_{q}=1.8$. We compute $d_{\min}\approx 0.82$ and $d_{\max}\approx 1.18$. The bounds are loose because *M* is relatively small.

We derive more properties of d(.) for the truncated Gaussian q(.). The derivative of (38) for x>0 is

(41) $$\begin{array}{c} d^{\prime }\left(x\right)=\sum_{k=-\lfloor M/2\rfloor }^{\lceil M/2\rceil -1}\frac{-(x+k\kappa )\kappa }{\sqrt{2\pi }c\sigma_{q}^{3}}e^{-{\left(x+k\kappa \right)}^{2}/\left(2\sigma_{q}^{2}\right)} \end{array}$$

and, by symmetry, we have $d^{\prime }\left(x\right)=-d^{\prime }\left(-x\right)$. Consider first odd *M* and x=0; the positive and negative summands cancel and $d^{\prime }\left(0\right)=0$; see Figure 2. For even *M*, there is a summand at k=−M/2 but not at k=M/2. Thus, $d^{\prime }\left(x\right)>0$ for small positive *x*. By symmetry, we thus have $d^{\prime }\left(x\right)<0$ for small negative *x*; see Figure 2.

## 5. Proof of Theorem 1

We use the definitions (33) and (34). Observe that we have

(42) $$\begin{array}{c} P_{X}=\frac{1}{2},\;P_{Z}=\frac{1}{2\sigma_{k,i}^{2}},\;\alpha =\frac{\sigma_{k,i}^{2}}{1+\sigma_{k,i}^{2}}. \end{array}$$

The achievable rate of sub-channel k,i is [62] (Equation (5))

(43) $$\begin{array}{cc} R_{k,i} & =I\left(U;Y^{\prime }\right)-I\left(U;S^{\prime }\right)\\ \; & =h\left(Y^{\prime }\right)-h\left(Z^{\prime }\;\mathrm{mod}\;A\right)-h\left(S^{\prime }\right)+h\left(X\right). \end{array}$$

We anticipate the result $R_{k,i}\approx \frac{1}{2}\log\left(1+\sigma_{k,i}^{2}\right)$ as follows. Section 5.2 shows that $h(Y^{\prime })\approx \log A$, which cancels $h(S^{\prime })=\log A$ for large *M*. Section 5.1 shows that $h\left(X\right)\approx \frac{1}{2}\log\left(\pi e\right)$ if *M* is large and $\sigma_{q}^{2}\approx 1/2$ and c≈1. Section 5.3 shows that the latter two approximations are valid for large *A*. Finally, we have the upper bound

(44) $$\begin{array}{cc} h(Z^{\prime }\;\mathrm{mod}\;A)\; & \overset{\left(a\right)}{\leq }\frac{1}{2}\log\left(2\pi e\;E\left[{\left(Z^{\prime }\;\mathrm{mod}\;A\right)}^{2}\right]\right)\\ \; & \leq \frac{1}{2}\log\left(2\pi e\;E\left[{\left(Z^{\prime }\right)}^{2}\right]\right)\\ \; & \overset{\left(b\right)}{=}\frac{1}{2}\log\left(\frac{\pi e}{1+\sigma_{k,i}^{2}}\right). \end{array}$$

Step (a) follows because a zero-mean Gaussian maximizes entropy under a second-moment constraint, and step (b) follows from $\sigma_{Z^{\prime }}^{2}=\alpha P_{Z}$; see (15) and (42).

The following two sections first study $P_{X}$ and h(X) and then $p(y^{\prime })$ and $h(Y^{\prime })$.

### 5.1. Power and Differential Entropy of X

The power $P_{X}=E\left[X^{2}\right]$ is based on an expectation with respect to p(.). Using (2) and (40), for symmetric unimodal q(.), we have

(45) $$\begin{array}{c} \frac{E_{q}\left[X^{2}\right]}{d_{\max}}\leq P_{X}\leq \frac{E_{q}\left[X^{2}\right]}{d_{\min}} \end{array}$$

and thus $P_{X}\to E_{q}\left[X^{2}\right]$ for M→∞. Using (40), we have (see Appendix C)

(46a) $$\begin{array}{cc} h(X) & \geq \frac{h_{q}\left(X\right)}{d_{\max}}+\log d_{\min}-\left(\frac{1}{d_{\min}}-\frac{1}{d_{\max}}\right){\left[q(0)\log q(0)\right]}^{+} \end{array}$$

(46b) $$\begin{array}{cc} h(X) & \leq \frac{h_{q}\left(X\right)}{d_{\min}}+\log d_{\max}+\left(\frac{1}{d_{\min}}-\frac{1}{d_{\max}}\right){\left[q(0)\log q(0)\right]}^{+} \end{array}$$

and for M→∞, we have (see (20))

(47) $$\begin{array}{c} h\left(X\right)\to h_{q}\left(X\right)=\frac{1}{2}\log\left(2\pi e\;\sigma_{q}^{2}c^{2}\right)-\frac{1}{2}\left(1-\frac{1}{2\sigma_{q}^{2}}\right)\log e. \end{array}$$

### 5.2. Density and Differential Entropy of $Y^{\prime }$

Consider $Y^{\prime }=\left(U+Z^{\prime }\right)\;\mathrm{mod}\;A$ in (14) where *U* and $Z^{\prime }=\left(\alpha -1\right)X+\alpha Z$ in (15) are independent. Note that *Z* is the sum of one Gaussian and several shaped random variables; see (31) and (33).

**Figure 4:**
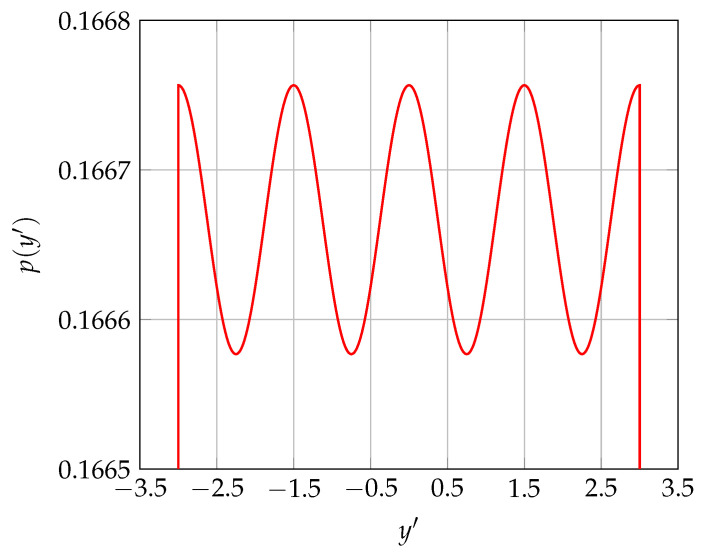
$p(y^{\prime })$ for A=6, M=4, $\sigma_{q}=1.8$, σ=1, $\tilde{Z}$ Gaussian and $P_{\tilde{Z}}=P_{X}$. Observe that $p(y^{\prime })$ is symmetric and periodic with period κ=1.5 and $p(y^{\prime })\approx 1/A$ in [−A/2,A/2).

Suppose *M* is even; the case where *M* is odd can be treated similarly. Using symmetric shaping for each sub-channel and Lemma 1, it follows that $p\left(z^{\prime }\right)=p\left(-z^{\prime }\right)$ and

$$\begin{array}{cc} p(y^{\prime }) & =\sum_{\ell \in Z}\sum_{v\in U}\frac{1}{M}p_{Z^{\prime }}\left(y^{\prime }-v-\ell A\right) \end{array}$$

(48a) $$\begin{array}{cc} \; & =\sum_{k\in Z}\frac{1}{M}p_{Z^{\prime }}\left(\left(y^{\prime }\;\mathrm{mod}\;\kappa \right)-\left(\frac{\kappa }{2}-k\kappa \right)\right) \end{array}$$

(48b) $$\begin{array}{cc} \; & =p_{Y^{\prime }}\left(y^{\prime }\;\mathrm{mod}\;\kappa \right) \end{array}$$

for $y^{\prime }\in \left[-A/2,A/2\right)$ and $p(y^{\prime })=0$ otherwise. Hence, $p(y^{\prime })$ is symmetric and periodic with period κ in [−A/2,A/2). Figure 4 illustrates these results for a point-to-point channel with A=6, M=4, $\sigma_{q}=1.8$, σ=1, $\tilde{Z}$ Gaussian, and $P_{\tilde{Z}}=P_{X}$. We show next that $p(y^{\prime })\approx 1/A$ in [−A/2,A/2) if *M* is large. In fact, increasing *M* to 8 in Figure 4 already gives $|p\left(y^{\prime }\right)-1/6|\leq 10^{-10}$.

The density $p(z^{\prime })$ is the convolution of three classes of densities, namely those of

- $\left(\alpha_{k,i}-1\right)X_{k,i}\;$;
- the $\alpha_{k,i}\;a_{k,i,l,h}X_{l,h}$ for l>k and all *h*;
- the $\alpha_{k,i}\;b_{k,i,h}W_{k,h}$ for all *h*.

The densities $q_{k,i}\left(.\right)$ and $q_{l,h}\left(.\right)$ are chosen to be symmetric unimodal, but the densities of $X_{k,i}$ and $X_{l,h}$ are not necessarily unimodal. However, the latter densities can each be lower-bounded by appropriate $q_{k,i}\left(.\right)/d_{\max}$ and $q_{l,h}\left(.\right)/d_{\max}$ as in (40), and these bounds are symmetric unimodal. Moreover, the Gaussian densities of the $W_{k,h}$ are symmetric unimodal. Thus, by Lemma 2 and using (39) and (40), we can lower bound $p_{Z^{\prime }}\left(.\right)$ by a symmetric unimodal $\underline{p}\left(.\right)$ that approaches $p_{Z^{\prime }}\left(.\right)$ for large *M*. Similarly, we can upper bound $p_{Z^{\prime }}\left(.\right)$ by a symmetric unimodal $\bar{p}\left(.\right)$ that approaches $p_{Z^{\prime }}\left(.\right)$ for large *M*. That is, we obtain

(49) $$\begin{array}{c} \underline{p}\left(z^{\prime }\right)\leq p\left(z^{\prime }\right)\leq \bar{p}\left(z^{\prime }\right) \end{array}$$

for symmetric unimodal $\underline{p}\left(z^{\prime }\right)$ and $\bar{p}\left(z^{\prime }\right)$ that both converge to $p(z^{\prime })$ for large *M*.

For example, point-to-point DPC has the effective noise $Z^{\prime }=\left(\alpha -1\right)X+\alpha Z$ where *X* and *Z* are independent; see (15). We hence have the convolution

(50) $$\begin{array}{c} p_{Z^{\prime }}\left(.\right)=p_{(\alpha -1)X}*p_{\alpha Z}\left(.\right). \end{array}$$

Note that p(x) is symmetric but not necessarily unimodal, and hence $p(z^{\prime })$ is symmetric but not necessarily unimodal. However, we may bound

(51) $$\begin{array}{cc} p(z^{\prime }) & =\int_{-A/2}^{A/2}p\left(x\right)\;p\left(z^{\prime }|x\right)\;dx\\ \; & \overset{\left(a\right)}{\geq }\int_{-A/2}^{A/2}\frac{q(x)}{d_{\max}}\;p\left(z^{\prime }|x\right)\;dx\\ \; & \overset{\left(b\right)}{=}\frac{1}{d_{\max}}q_{(\alpha -1)X}*p_{\alpha Z}\left(z^{\prime }\right):=\underline{p}\left(z^{\prime }\right) \end{array}$$

where step (a) follows by (40) and step (b) uses the density

(52) $$\begin{array}{c} q_{(\alpha -1)X}\left(x\right)=\frac{q(x/(\alpha -1))}{1-\alpha } \end{array}$$

which is symmetric unimodal in *x*. The function $\underline{p}\left(z^{\prime }\right)$ defined in (51) is a convolution of two symmetric unimodal functions. Thus, $\underline{p}\left(z^{\prime }\right)$ is symmetric unimodal by Lemma 2. Moreover, one can iterate the above argument for successive DPC with K>1, and thereby obtain a symmetric unimodal $\underline{p}\left(z^{\prime }\right)$ with $\underline{p}\left(z^{\prime }\right)\leq p\left(z^{\prime }\right)$.

**Figure 5:**
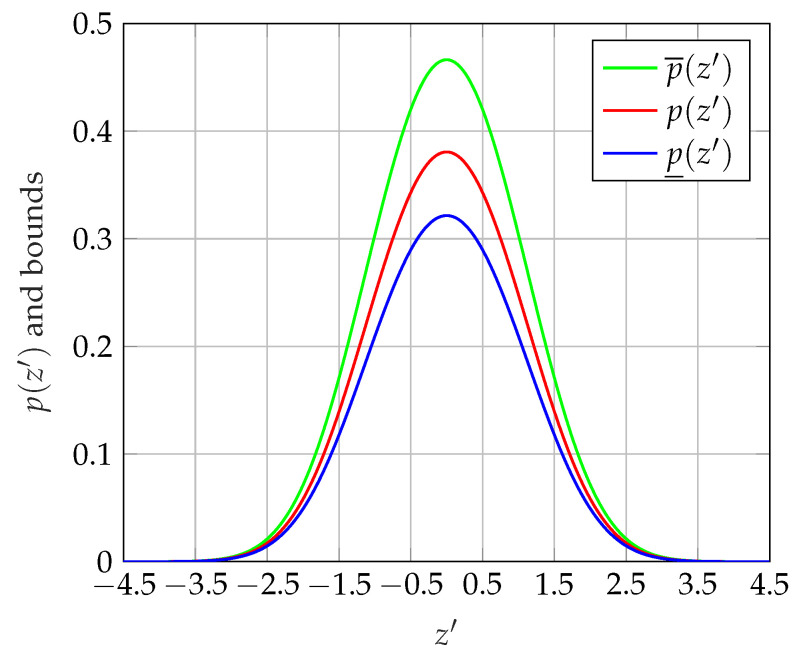
$p(z^{\prime })$ and bounds for A=6, M=8, and truncated Gaussian shaping.

Let $\bar{p}\left(z^{\prime }\right)$ be the same as $\underline{p}\left(z^{\prime }\right)$ but with $d_{\min}$ replacing $d_{\max}$ in (51). We have the bounds (49) where both $\underline{p}\left(z^{\prime }\right)$ and $\bar{p}\left(z^{\prime }\right)$ are symmetric unimodal. Figure 5 shows the curves for A=6, M=8, the truncated Gaussian q(.) with $\sigma_{q}=1.8$, σ=1, $P_{X}=P_{Z}$, and *Z* is Gaussian.

Now substitute ${\underline{p}}_{Z^{\prime }}\left(.\right)$ and ${\bar{p}}_{Z^{\prime }}\left(.\right)$ for f(.) in (8) of Lemma 3 to write

(53a) $$\begin{array}{c} -\kappa \;{\underline{p}}_{Z^{\prime }}\left(0\right)\leq \frac{1}{d_{\max}}-\sum_{k\in Z}\kappa \;{\underline{p}}_{Z^{\prime }}\;\left(y^{\prime }+k\kappa \right)\leq \kappa \;{\underline{p}}_{Z^{\prime }}\left(0\right) \end{array}$$

(53b) $$\begin{array}{c} -\kappa \;{\bar{p}}_{Z^{\prime }}\left(0\right)\leq \frac{1}{d_{\min}}-\sum_{k\in Z}\kappa \;{\bar{p}}_{Z^{\prime }}\;\left(y^{\prime }+k\kappa \right)\leq \kappa \;{\bar{p}}_{Z^{\prime }}\left(0\right) \end{array}$$

for any $y^{\prime }$. Define the expressions

(54a) $$\begin{array}{cc} p_{\min}\; & =\frac{1}{A}\left(\frac{1}{d_{\max}}-\kappa \;{\underline{p}}_{Z^{\prime }}\left(0\right)\right) \end{array}$$

(54b) $$\begin{array}{cc} p_{\max} & =\frac{1}{A}\left(\frac{1}{d_{\min}}+\kappa \;{\bar{p}}_{Z^{\prime }}\left(0\right)\right). \end{array}$$

Applying (53) gives

(55) $$\begin{array}{cc} p_{\min} & \leq \sum_{k\in Z}\frac{1}{M}{\underline{p}}_{Z^{\prime }}\left(y^{\prime }+k\kappa \right)\overset{\left(a\right)}{\leq }p\left(y^{\prime }\right)\overset{\left(b\right)}{\leq }\sum_{k\in Z}\frac{1}{M}{\bar{p}}_{Z^{\prime }}\left(y^{\prime }+k\kappa \right)\leq p_{\max} \end{array}$$

for $y^{\prime }\in \left[-A/2,A/2\right)$, where steps (a) and (b) follow by (48a) and (49). For example, for the parameters in Figure 4, we have $p_{\min}\approx 0.1$ and $p_{\max}\approx 0.26$. The bounds are again loose because *M* is small. However, as M→∞, we have $p_{\min},p_{\max}\to 1/A$, and hence $p(y^{\prime })$ converges to the uniform distribution over [−A/2,A/2). Finally, assuming *M* is sufficiently large such that $p_{\min}>0$, we obtain

(56) $$\begin{array}{c} -\log p_{\max}\leq h\left(Y^{\prime }\right)\leq -\log p_{\min} \end{array}$$

which implies $h(Y^{\prime })\to \log A$ for M→∞.

### 5.3. Achieving Capacity

For fixed *A*, $\sigma_{q}$ satisfying $E_{q}\left[X^{2}\right]=1/2$, and M→∞, we obtain the bound (35) by using (44), (47) and (56). Finally, consider three possible scalings as A→∞:

- $A/\sigma_{q}\to \infty$: We have $c=1-2Q\left(A/\left(2\sigma_{q}\right)\right)\to 1$ and $\sigma_{q}^{2}\to 1/2$. We thus obtain the desired (36).
- $A/\sigma_{q}\to b$ for a constant *b*: This is impossible because $\sigma_{q}^{2}\to \infty$ as A→∞, which forces $P_{X}\to \infty$, contradicting the power constraint.
- $A/\sigma_{q}\to 0$: We have $c=1-2Q\left(A/\left(2\sigma_{q}\right)\right)\to 0$. Consider the Taylor expansions around x=0:
  (57) $$\begin{array}{cc} Q(x/2) & =\frac{1}{2}-\frac{x}{2\sqrt{2\pi }}+\frac{x^{3}}{48\sqrt{2\pi }}+O\left(x^{5}\right) \end{array}$$
  (58) $$\begin{array}{cc} e^{-x^{2}/8} & =1-x^{2}/8+x^{4}/128+O\left(x^{6}\right). \end{array}$$
  From (57), we obtain
  (59) $$\begin{array}{c} c=\frac{1}{\sqrt{2\pi }}\frac{A}{\sigma_{q}}-\frac{1}{24\sqrt{2\pi }}{\left(\frac{A}{\sigma_{q}}\right)}^{3}+O\left({\left(\frac{A}{\sigma_{q}}\right)}^{5}\right). \end{array}$$
  Applying (57)–(59) to (2), we have
  (60) $$\begin{array}{cc} E[X^{2}]\; & =\lim_{A/\sigma_{q}\to 0}\sigma_{q}^{2}\left[1-\frac{1-\frac{1}{8}{\left(\frac{A}{\sigma_{q}}\right)}^{2}+\frac{1}{128}{\left(\frac{A}{\sigma_{q}}\right)}^{4}}{1-\frac{1}{24}{\left(\frac{A}{\sigma_{q}}\right)}^{2}}\right]\\ \; & =\frac{A^{2}}{12}+\lim_{A/\sigma_{q}\to 0}\sigma_{q}^{2}\;O\left({\left(\frac{A}{\sigma_{q}}\right)}^{4}\right). \end{array}$$
  Thus, we have $E[X^{2}]\to \infty$ for A→∞, contradicting the power constraint.

## 6. Simulation Models and Capacity

Consider a BS serving K=2 UEs, each with $n_{k}=1$ antenna and unit-variance noise. We have ${\underline{X}}_{k}=P_{k}{\tilde{X}}_{k}$ and

(61a) $$\begin{array}{cc} Y_{1} & =H_{1}{\underline{X}}_{1}+H_{1}{\underline{X}}_{2}+Z_{1} \end{array}$$

(61b) $$\begin{array}{cc} Y_{2} & =H_{2}{\underline{X}}_{1}+H_{2}{\underline{X}}_{2}+Z_{2} \end{array}$$

where each $H_{k}$ is a row vector of dimension $n_{t}$, and ${∥P_{1}∥}^{2}+{∥P_{2}∥}^{2}\leq P_{X}$.

### 6.1. DPC and Duality

The DPC rate bounds (22) simplify to

(62a) $$\begin{array}{cc} R_{1} & \leq \log\left(1+\frac{|H_{1}P_{1}{|}^{2}}{1+|H_{1}P_{2}{|}^{2}}\right) \end{array}$$

(62b) $$\begin{array}{cc} R_{2} & \leq \log\left(1+|H_{2}P_{2}{|}^{2}\right). \end{array}$$

The optimal precoders $P_{k}$ can be found by using the dual MAC with output

(63) $$\begin{array}{c} \underline{Y}=\left[\begin{array}{c} Y_{1}\\ Y_{2} \end{array}\right]=H_{1}^{†}X_{1}+H_{2}^{†}X_{2}+\underline{Z} \end{array}$$

and independent inputs $X_{1}$, $X_{2}$ with variances $P_{1}$, $P_{2}$ that satisfy $P_{1}+P_{2}\leq P_{X}$; see [41]. The noise $\underline{Z}={\left[Z_{1}\;Z_{2}\right]}^{T}$ has i.i.d. entries with unit variance. Let ${\bar{P}}_{1}^{†}$ and ${\bar{P}}_{2}^{†}$ be MAC receiver beamforming vectors that create the scalar outputs

(64) $$\begin{array}{c} {\bar{Y}}_{1}={\bar{P}}_{1}^{†}\underline{Y},\;{\bar{Y}}_{2}={\bar{P}}_{2}^{†}\underline{Y} \end{array}$$

for UEs 1 and 2, respectively. The optimal ${\bar{P}}_{k}$ are specified by LMMSE estimation; see Appendix D. It remains to determine the powers $P_{1}$ and $P_{2}$, which is a concave optimization problem in general.

Next, following [44,45], we choose the BC precoding vectors as co-linear with the ${\bar{P}}_{k}$:

(65) $$\begin{array}{c} P_{1}=a_{1}\;{\bar{P}}_{1},\;P_{2}=a_{2}\;{\bar{P}}_{2} \end{array}$$

for real scalars $a_{1},a_{2}$. Suppose the BC first encodes for UE 1, and the MAC receiver first decodes the UE 2 signal. For the desired rate equivalences, we require the signal-to-interference-and-noise ratios (SINRs) to satisfy

(66a) $$\begin{array}{cc} \; & \frac{|H_{1}P_{1}{|}^{2}}{1+|H_{1}P_{2}{|}^{2}}=\frac{|P_{1}^{†}H_{1}^{†}{|}^{2}P_{1}/a_{1}^{2}}{{∥P_{1}∥}^{2}/a_{1}^{2}} \end{array}$$

(66b) $$\begin{array}{cc} \; & |H_{2}P_{2}{|}^{2}=\frac{|P_{2}^{†}H_{2}^{†}{|}^{2}P_{2}/a_{2}^{2}}{{∥P_{2}∥}^{2}/a_{2}^{2}+|P_{2}^{†}H_{1}^{†}{|}^{2}P_{1}/a_{2}^{2}} \end{array}$$

where the left- and right-hand sides are BC and MAC SINRs, respectively. Using $|H_{k}P_{l}{|}^{2}={\left|P_{l}^{†}H_{k}^{†}\right|}^{2}$, we obtain

(67a) $$\begin{array}{cc} P_{1} & =\frac{∥P_{1}{∥}^{2}}{1+|H_{1}P_{2}{|}^{2}} \end{array}$$

(67b) $$\begin{array}{cc} P_{2} & =∥P_{2}{∥}^{2}+\frac{|H_{1}P_{2}{|}^{2}}{1+|H_{1}P_{2}{|}^{2}}{∥P_{1}∥}^{2} \end{array}$$

and thus

(68) $$\begin{array}{c} P_{1}+P_{2}=∥P_{1}{∥}^{2}+{∥P_{2}∥}^{2}. \end{array}$$

**Figure 6:**
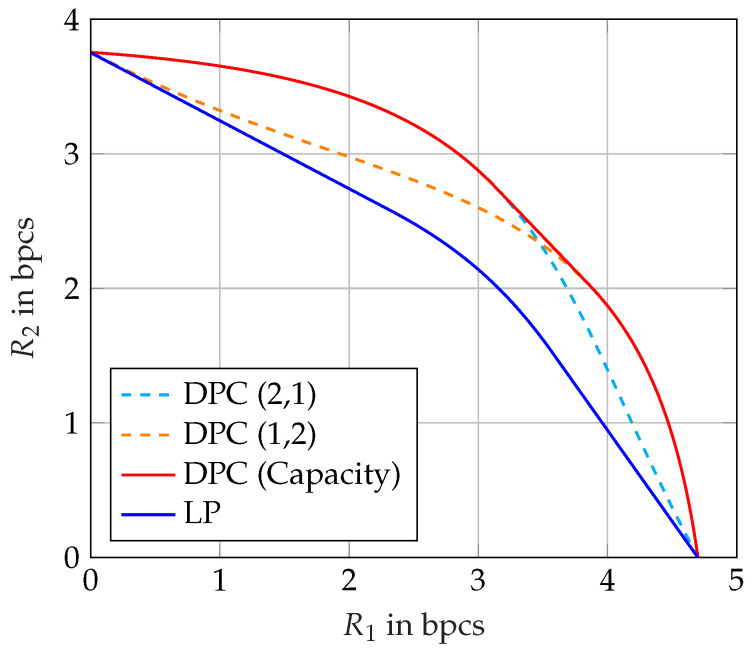
DPC and LP rate regions for the channels (69) with $h_{1}=1$, $h_{2}=0.5$ and $P_{X}=25$.

For example, consider $P_{X}=25$ and the channel vectors

(69) $$\begin{array}{c} H_{1}=h_{1}[1\;0],\;\;\;H_{2}=h_{2}[1\;1] \end{array}$$

with $h_{1}=1$, $h_{2}=0.5$. We use the dual MAC to compute the capacity region shown in Figure 6, where the rates are measured in bits per channel symbol (bpcs). This region is the union of two regions corresponding to the two encoding orders, shown as dashed lines.

### 6.2. Coded LP

We compare with linear precoding (LP) where the transmit vector is $X=\sum_{k=1}^{K}X_{k}$ with independent $X_{k}$. The bound (22) becomes more restrictive:

(70) $$\begin{array}{c} R_{k}\leq \log\det\left(I+H_{k}K_{k}H_{k}^{†}{\left(Q_{k}+\sum_{l\neq k}H_{k}K_{l}H_{k}^{†}\right)}^{-1}\right) \end{array}$$

where the sum inside the inverse is now over l≠k. For example, for K=2, $n_{k}=1$, and unit-variance noise, the bounds are (see (62))

(71a) $$\begin{array}{cc} R_{1} & \leq \log\left(1+\frac{|H_{1}P_{1}{|}^{2}}{1+|H_{1}P_{2}{|}^{2}}\right) \end{array}$$

(71b) $$\begin{array}{cc} R_{2} & \leq \log\left(1+\frac{|H_{2}P_{2}{|}^{2}}{1+|H_{2}P_{1}{|}^{2}}\right). \end{array}$$

Appendix E reviews a BC-MAC duality for LP. The optimal beamforming vectors ${\bar{P}}_{k}$ in (64) for the dual MAC are again specified by LMMSE estimation, and we may again use the relations (65); see [44]. Unfortunately, unlike DPC, the power allocation problem is no longer concave. We thus perform an exhaustive search over a fine quantization of power allocations, which is possible for small numbers of UEs and antennas. The LP region for the channels (69) with $h_{1}=1$, $h_{2}=0.5$, and $P_{X}=25$ is shown in Figure 6.

We remark that LP can be improved, see Section 1.1. For example, one may use multiple transmission modes, rate splitting, and SIC; see Appendixes Appendix F and Appendix G. However, rate splitting and SIC increase UE complexity and latency.

### 6.3. Parallel Channels

We study parallel channels that correspond to OFDM tones. A BC with two transmit antennas, one antenna per UE, and two tones may be written as having $n_{t}=4$ and $n_{1}=n_{2}=2$, where the rows of each $H_{k}$ are orthogonal. For example, consider

(72a) H1=0.10000.0.000.800.

(72b) H2=0.0.50.5000.0.000.60.60.

where the first and second rows of $H_{1}$ and $H_{2}$ describe the channels of tones 1 and 2, respectively. The tone channels may equivalently be described by the matrices

Htone1=0.100.0.0.50.50.,Htone2=0.0.800.0.0.60.60.

where the first and second rows are the channels of receivers 1 and 2, respectively.

For DPC, we use the dual MAC and optimize weighted sum rates $w_{1}R_{1}+w_{2}R_{2}$ over

- The power allocation across the parallel MACs;
- The users’ transmit powers for the two MACs;
- The MAC decoding orders.

One can apply any convex solver, e.g., gradient descent [89]. For LP, one may consider either the BC or dual MAC and optimize the weighted sum rate over

- The power allocation across the parallel MACs;
- The users’ transmit powers for the two MACs.

We use the dual MAC and LMMSE estimation, see Section 6.2, and perform an exhaustive search over a fine quantization of power allocations. For problems with more UEs and antennas, one must resort to other methods, e.g., gradient-based methods as in [90].

**Figure 7:**
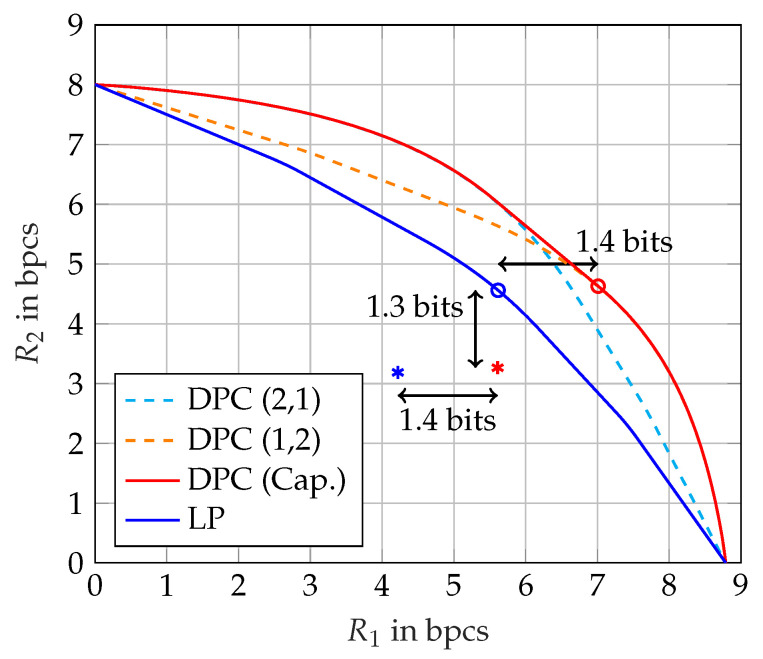
DPC and LP rate regions for the channels (72) and $P_{X}=50$. Circles show points that maximize the sum rate. Asterisks show points achieved with blocks of 64 complex QAM symbols per tone, multi-level polar codes with 256 bits per level, and a target $\mathrm{FER}=10^{-3}$ per user.

The capacity and LP rate regions are shown in Figure 7 for the transmit power $P_{X}=50$. The dashed lines indicate the DPC regions for the two encoding orders. The circles show points $\left(R_{1},R_{2}\right)$ that maximize the sum rates, namely

(73a) $$\begin{array}{cc} \mathrm{DPC}:\; & \left(R_{1},R_{2}\right)\approx \left(7.01,4.63\right)\;\Rightarrow \;R_{1}+R_{2}=11.64 \end{array}$$

(73b) $$\begin{array}{cc} \mathrm{LP}:\; & \left(R_{1},R_{2}\right)\approx \left(5.62,4.56\right)\;\Rightarrow \;R_{1}+R_{2}=10.18. \end{array}$$

The asterisks show points achieved by optimizing multi-level polar codes with 256 bits per level at the target $\mathrm{FER}=10^{-3}$. The result is (see Section 7.3 and Table 1 below)

(74a) $$\begin{array}{cc} \mathrm{DPC}:\; & \left(R_{1},R_{2}\right)\approx \left(5.61,3.27\right)\;\Rightarrow \;R_{1}+R_{2}=8.88 \end{array}$$

(74b) $$\begin{array}{cc} \mathrm{LP}:\; & \left(R_{1},R_{2}\right)\approx \left(4.22,3.19\right)\;\Rightarrow \;R_{1}+R_{2}=7.41. \end{array}$$

### 6.4. Polar Code Simulations

We use the channel (72) with $P_{X}=50$ to perform polar code simulations described in Section 7. We target the DPC rate pair in (73) via the DPC order 1,2, and apply the steps in Section 3.1. The resulting singular values for the sub-channels (29) are

(75a) $$\begin{array}{cc} \mathrm{UE}1: & \;\;\sigma_{1,1}\approx 3.6675,\;\;\sigma_{1,2}\approx 2.8131 \end{array}$$

(75b) $$\begin{array}{cc} \mathrm{UE}2: & \;\;\sigma_{2,1}\approx 2.2552,\;\;\sigma_{2,2}\approx 1.7519. \end{array}$$

For the LP rate pair in (73), the optimized precoders $P_{k}$ are used to compute the covariance matrices $\Sigma_{k}$, and then the steps in Section 3.1 are used to compute the singular values with no interference ${\underline{S}}_{1}={\underline{S}}_{2}=\underline{0}$ and enhanced noise

(76) $$\begin{array}{c} {\underline{\overset{ˇ}{Z}}}_{1}=H_{1}{\underline{X}}_{2}+{\underline{Z}}_{1},\;{\underline{\overset{ˇ}{Z}}}_{2}=H_{2}{\underline{X}}_{1}+{\underline{Z}}_{2}. \end{array}$$

The resulting singular values for the best sum rate are

(77a) $$\begin{array}{cc} \mathrm{UE}1: & \;\;\sigma_{1,1}\approx 2.8635,\;\;\sigma_{1,2}\approx 2.0786 \end{array}$$

(77b) $$\begin{array}{cc} \mathrm{UE}2: & \;\;\sigma_{2,1}\approx 2.2722,\;\;\sigma_{2,2}\approx 1.6843. \end{array}$$

## 7. Short Polar Codes

**Figure 8:**
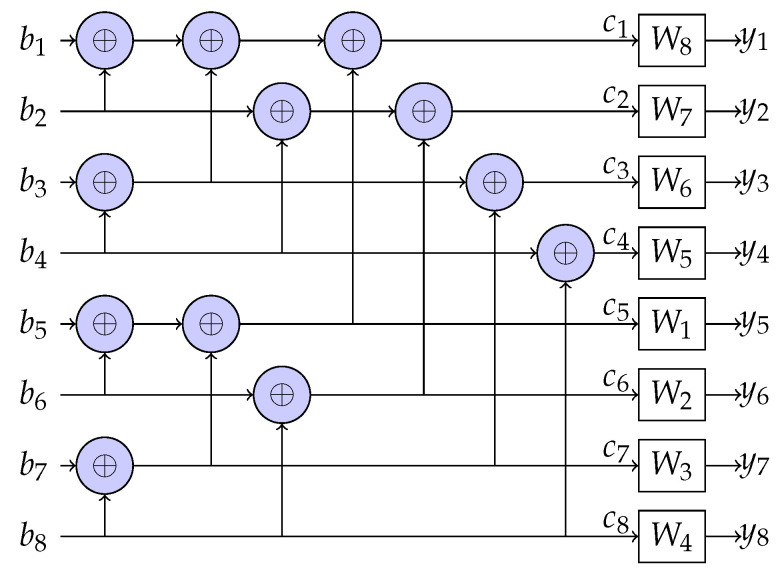
Polar encoding for N=8.

Let $N=2^{n}$ for a positive integer *n*. The N×N polar encoding matrix is

(78) $$\begin{array}{c} G_{N}={\left[\begin{array}{cc} 1 & 0\\ 1 & 1 \end{array}\right]}^{⊗n},\;n=\log_{2}N. \end{array}$$

where ⊗n denotes an *n*-fold Kronecker product. An *N*-dimensional bit vector $\underline{b}$ is encoded as ${\underline{c}}^{T}={\underline{b}}^{T}G_{N}$. The recursive block structure of $G_{N}$ yields

(79) $$\begin{array}{cc} {\underline{c}}^{T} & ={\underline{b}}^{T}\left[\begin{array}{cc} G_{N/2} & 0\\ G_{N/2} & G_{N/2} \end{array}\right]\\ \; & =\left[\underset{{\underline{c}}_{1}^{T}}{\underset{︸}{{\underline{b}}_{1:N/2}^{T}G_{N/2}⊕{\underline{b}}_{N/2+1:N}^{T}G_{N/2}}},\;\;\underset{{\underline{c}}_{2}^{T}}{\underset{︸}{{\underline{b}}_{N/2+1:N}^{T}G_{N/2}}}\right] \end{array}$$

and thus, ${\underline{b}}_{1:N/2}^{T}G_{N/2}={\underline{c}}_{1}^{T}⊕{\underline{c}}_{2}^{T}$ and ${\underline{c}}_{2}^{T}$ are the codewords obtained by encoding the first and second halves of ${\underline{b}}^{T}$, respectively. Thus, ${\underline{c}}^{T}$ is constructed by first encoding the sub-blocks ${\underline{b}}_{1:N/2}^{T}$ and ${\underline{b}}_{N/2+1:N}^{T}$, and then applying a polarization transform to combine these subcodes. For example, Figure 8 shows that the encoder can first map ${\underline{b}}_{1:4}$ and ${\underline{b}}_{5:8}$ to ${\underline{c}}_{1:4}⊕{\underline{c}}_{5:8}$ and ${\underline{c}}_{5:8}$, respectively, and then apply one level of the polarization transform (n=1) to the corresponding sub-codeword elements. We exploit this structure to construct a long polar code across two sub-channels of a UE by using two separate polar subcodes.

### 7.1. Shaped Multi-Level Coding

There are two variants of shaping with polar codes: all coded bits are assigned the same or different *a priori* distributions. The former approach applies to time-invariant coding, and the latter to time-varying coding. An example of a time-varying coding is DPC, because the interference varies with each coded bit.

We use shaped multi-level coding (MLC) to map message bits to ASK symbols. A simple approach uses separate polar codes for each sub-channel. However, at short block lengths, performance improves by using a single, long multi-level polar code across all sub-channels of a user.

Another effective approach leverages the different singular values in (29), to enhance polarization [63,75,91]. Let $I(W_{i})$, i∈{1,…,N}, denote the mutual information of the polar code bit channels, ordered such that $I\left(W_{i}\right)\leq I\left(W_{j}\right)$ for i≤j. Further, let ${\underline{W}}^{-}$ and ${\underline{W}}^{+}$ denote the indices of the least reliable N/2 and most reliable N/2 code bit channels, respectively, with ${\underline{W}}^{-}$ in ascending order and ${\underline{W}}^{+}$ in descending order. By matching the corresponding elements of ${\underline{W}}^{+}$ and ${\underline{W}}^{-}$ in the last polarization stage, the FER performance of the polar code can be improved. For example, in Figure 8, the code bits transmitted on $W_{1},W_{2},W_{3},W_{4}$ are matched in the last polarization stage with the code bits transmitted on $W_{8},W_{7},W_{6},W_{5}$, respectively.

### 7.2. Polar-Coded Modulation

The BS sends 64 symbols $\underline{x}\left(t\right)$, t=1,…,64. Alternatively, for our example channel (72), it sends 64 symbols over each of the $n_{\mathrm{ch}}=4$ parallel channels with inputs

$$\begin{array}{cc} \; & {\tilde{x}}_{1,1}\left(t\right),\;{\tilde{x}}_{1,2}\left(t\right)\;\mathrm{for}\;\mathrm{UE}\;1\\ \; & {\tilde{x}}_{2,1}\left(t\right),\;{\tilde{x}}_{2,2}\left(t\right)\;\mathrm{for}\;\mathrm{UE}\;2. \end{array}$$

We choose the ${\tilde{x}}_{1,1}\left(t\right)$ and ${\tilde{x}}_{1,2}\left(t\right)$ in DPC to have a 256-QAM alphabet, while all other ${\tilde{x}}_{k,i}\left(t\right)$ have a 64-QAM alphabet. The reason for choosing a larger QAM alphabet for DPC and UE 1 is that we selected a particular shaping parameter ν in Section 7.3 for which 16-ASK is required to achieve rates close to the AWGN channel capacity.

These alphabets are split into two real alphabets: 16-ASK and 8-ASK. There are, therefore, 128 real symbols ${\tilde{x}}_{k,i,R}\left(t^{\prime }\right)$ for fixed i,k and $t^{\prime }=1,\ldots ,128$. We map these 128 symbols to the complex QAM symbols via

(80) $$\begin{array}{c} {\tilde{x}}_{k,i}\left(t\right)={\tilde{x}}_{k,i,R}\left(2t-1\right)+j\;{\tilde{x}}_{k,i,R}\left(2t\right) \end{array}$$

for t=1,…,64. The precoded transmit vectors are

(81) $$\begin{array}{c} {\underline{x}}_{k}\left(t\right)=P_{k}\left[\begin{array}{c} {\tilde{x}}_{k,1}\left(t\right)\\ {\tilde{x}}_{k,2}\left(t\right) \end{array}\right] \end{array}$$

for k=1,2 and t=1,…,64. The BS in DPC thus sends $64\cdot \log_{2}\left(256\right)=512$ and $64\cdot \log_{2}\left(64\right)=384$ coded bits over the sub-channels of UE 1 and UE 2, respectively, corresponding to a total of 1024 and 768 coded bits for UE 1 and UE 2, respectively. The BS uses MLC with 4 or 3 levels across sub-channel pairs, and each level has 256 coded bits.

Consider DPC with the precoding order k=1,2. The message bits of UE 1 are encoded directly into the 256 real 16-ASK symbols in the order

$$\begin{array}{c} {\tilde{x}}_{1,1,R}\left(1\right),\ldots ,{\tilde{x}}_{1,1,R}\left(128\right),{\tilde{x}}_{1,2,R}\left(1\right),\ldots ,{\tilde{x}}_{1,2,R}\left(128\right) \end{array}$$

by using MLC with 4 levels and a length-256 polar code for each level. From (80) and (81), the first and second halves of the polar code bits protecting the bit levels of ${\tilde{\underline{x}}}_{1,R}$ are transmitted over sub-channels with channel coefficients $\sigma_{1,1}$ and $\sigma_{1,2}$, respectively; see (27).

Let 1 be the 128-dimensional vector with 128 ones. The encoding parameters for the two sub-channels of UE 2 can be interpreted as the 256-dimensional vectors

(82) $$\begin{array}{cc} {\underline{\alpha }}_{2} & ={\left[\alpha_{2,1},\alpha_{2,2}\right]}^{T}⊗1 \end{array}$$

(83) $$\begin{array}{cc} {\underline{A}}_{2} & ={\left[A_{2,1},A_{2,2}\right]}^{T}⊗1 \end{array}$$

(84) $$\begin{array}{cc} {\underline{\sigma }}_{{\tilde{X}}_{2}}^{2} & ={\left[\sigma_{{\tilde{X}}_{2,1}}^{2},\sigma_{{\tilde{X}}_{2,2}}^{2}\right]}^{T}⊗1 \end{array}$$

(85) $$\begin{array}{cc} {\underline{\sigma }}_{Z_{2}^{\prime }}^{2} & ={\left[\sigma_{Z_{2,1}^{\prime }}^{2},\sigma_{Z_{2,2}^{\prime }}^{2}\right]}^{T}⊗1. \end{array}$$

For UE 2, the BS treats ${\underline{\tilde{s}}}_{2}=F_{2}H_{2}{\underline{X}}_{1}$ as interference; see (29). To encode to 256 real 8-ASK symbols ${\underline{u}}_{2}$, the BS applies

(86) $$\begin{array}{c} s_{2,i,R}^{\prime }\left(t^{\prime }\right)=\left(\alpha_{2,i}{\tilde{s}}_{2,i,R}\left(t^{\prime }\right)+d_{2,i}\left(t^{\prime }\right)\right)\;\mathrm{mod}\;A_{2,i} \end{array}$$

for i=1,2 and $t^{\prime }=1,\ldots ,128$. To simplify notation, write the 256 coded bit indexes as i=1,…,256 and define

(87) $$\begin{array}{c} {\tilde{s}}_{2,R}\left(1\right),\ldots ,{\tilde{s}}_{2,R}\left(256\right):={\tilde{s}}_{2,1,R}\left(1\right),\ldots ,{\tilde{s}}_{2,1,R}\left(128\right),{\tilde{s}}_{2,2,R}\left(1\right),\ldots ,{\tilde{s}}_{2,2,R}\left(128\right). \end{array}$$

Note that we used the same ordering as above for ${\tilde{x}}_{1,i,R}\left(t^{\prime }\right)$. Similarly, write

(88) $$\begin{array}{c} s_{2,R}^{\prime }\left(1\right),\ldots ,s_{2,R}^{\prime }\left(256\right):=s_{2,1,R}^{\prime }\left(1\right),\ldots ,s_{2,1,R}^{\prime }\left(128\right),s_{2,2,R}^{\prime }\left(1\right),\ldots ,s_{2,2,R}^{\prime }\left(128\right). \end{array}$$

The BS performs shaping by computing the log-likelihood ratio (LLR)

(89) $$\begin{array}{cc} \; & L\left(c^{l}\left(i\right)|c^{1}\left(i\right),\ldots ,c^{l-1}\left(i\right),{\tilde{s}}_{2,R}\left(i\right)\right)=\\ \; & \frac{\sum_{u_{2}\in V_{2}\left(c^{l}\left(i\right)=0|c^{1}\left(i\right),\ldots ,c^{l-1}\left(i\right)\right)}\exp\left(\frac{-{\left(\left(u_{2}-s_{2,R}^{\prime }\left(i\right)\right)\;\mathrm{mod}\;A_{2}\left(i\right)\right)}^{2}}{2\sigma_{{\tilde{X}}_{2}}{\left(i\right)}^{2}}\right)}{\sum_{u_{2}\in V_{2}\left(c^{l}\left(i\right)=1|c^{1}\left(i\right),\ldots ,c^{l-1}\left(i\right)\right)}\exp\left(\frac{-{\left(\left(u_{2}-s_{2,R}^{\prime }\left(i\right)\right)\;\mathrm{mod}\;A_{2}\left(i\right)\right)}^{2}}{2\sigma_{{\tilde{X}}_{2}}{\left(i\right)}^{2}}\right)} \end{array}$$

of the coded bit $c^{l}\left(i\right)$ at the *l*th bit-level, i=1,…,256, l=1,2,3, where we used the shaping density (18) and similar notation as (82)–(85). A polar decoder treats both the frozen and message bits as frozen to compute the shaping bits. The set $V_{2}\left(c^{l}\left(i\right)|c^{1}\left(i\right),\ldots ,c^{l-1}\left(i\right)\right)$ in (89) is the subset of 8-ASK symbols for which the first *l* labeling bits are $c^{1}\left(i\right),\ldots ,c^{l}\left(i\right)$.

The encoding output is 256 real 8-ASK symbols in the order

$$\begin{array}{c} u_{2,1,R}\left(1\right),\ldots ,u_{2,1,R}\left(128\right),u_{2,2,R}\left(1\right),\ldots ,u_{2,2,R}\left(128\right). \end{array}$$

Using these symbols, the BS computes

(90) $$\begin{array}{c} {\tilde{x}}_{2,i,R}\left(t^{\prime }\right)=\left(u_{2,i,R}\left(t^{\prime }\right)-s_{2,i,R}^{\prime }\left(t^{\prime }\right)\right)\;\mathrm{mod}\;A_{2,i} \end{array}$$

for i=1,2 and $t^{\prime }=1,\ldots ,128$. These symbols are then mapped to the ${\tilde{x}}_{2,i}\left(t\right)$ according to (80), and to the ${\underline{x}}_{2}\left(t\right)$ according to (81).

To decode, the UEs compute the LLRs

(91) $$\begin{array}{cc} \; & L(c^{l}\left(i\right)|{\hat{c}}^{1}\left(i\right),\ldots ,{\hat{c}}^{l-1}\left(i\right),y^{\prime }\left(i\right))=\\ \; & \frac{\sum_{u_{2}\in V_{2}\left(c^{l}\left(i\right)=0|{\hat{c}}^{1}\left(i\right),\ldots ,{\hat{c}}^{l-1}\left(i\right)\right)}\sum_{m=-\infty }^{\infty }\exp\left(\frac{-{\left(y^{\prime }\left(i\right)-u_{2}\;+\;mA_{2}\left(i\right)\right)}^{2}}{2\sigma_{Z_{2}^{\prime }}{\left(i\right)}^{2}}\right)}{\sum_{u_{2}\in V_{2}\left(c^{l}\left(i\right)=1|{\hat{c}}^{1}\left(i\right),\ldots ,{\hat{c}}^{l-1}\left(i\right)\right)}\sum_{m=-\infty }^{\infty }\exp\left(\frac{-{\left(y^{\prime }\left(i\right)-u_{2}\;+\;mA_{2}\left(i\right)\right)}^{2}}{2\sigma_{Z_{2}^{\prime }}{\left(i\right)}^{2}}\right)} \end{array}$$

for their bits $c^{l}\left(i\right)$, where the inner sum in (91) is due to the modulo operator in (13).

### 7.3. Code Design

The modulo operator limits the dynamic range at the UEs and BS, especially in the presence of interference. However, the dispersion increases at low rates, and this increases the FER at short block length [62] (Figure 5 and Figure 6). We therefore use a modulo operator only for DPC; i.e., we do not use a modulo interval for UE 1 under DPC, or for either UE in the LP scheme. Instead, we shaped with a sampled Gaussian distribution proportional to $e^{-\nu x^{2}}$ with ν=0.494. The ASK spacings were κ=0.93 and 0.415 for M=8 and 16, respectively. For UE 2 with DPC, we cancel interference by shaping according to (12), and use A=6.64 and $\sigma_{q}=1$ for both sub-channels. The BS uses successive cancellation (SC) decoding for shaping. The UEs use SC list (SCL) decoding with list size 8 and list passing across subcodes and bit levels for channel decoding [71,92].

We allocate an asymptotically optimal fraction of sub-channels to shaping at each bit level j=1,2,3,4; see [60] (Section IV). For the two polar subcodes at each bit level, the mutual information is computed from this quantity and translated proportionally into the fraction of shaping bits assigned to each subcode. The most reliable sub-channels are allocated to the shaping bits, the next most reliable sub-channels to the message bits, and the remaining sub-channels to the frozen bits. The sub-channel reliability order specified in 5G NR [93] is used. We employ 6 CRC bits, which are transmitted on the subcode of the last bit level. At this level, after allocating the shaping bits, the most reliable remaining sub-channels are assigned to the CRC bits. Natural labeling maps bits to modulation symbols.

After allocating the shaping and CRC bits, the assignment of message bits to the bit levels and their subcodes is optimized to maximize the information rate at a target $\mathrm{FER}=10^{-3}$ for $P_{X}=50$. The resulting parameters are given in Table 1, where $s^{i}$ and $b^{i}$ are the numbers of shaping and message bits at bit level *i*, respectively, and “sub” indicates the subcode index. The parameters would make the variances of the ${\tilde{X}}_{k,i}$ slightly larger than 1 due to finite-block-length shaping. We thus normalized so that the ${\tilde{X}}_{k,i}$ have unit variance.

**Table 1:** Polar code parameters.

		Sub	$s^{1}$	$s^{2}$	$s^{3}$	$s^{4}$	$b^{1}$	$b^{2}$	$b^{3}$	$b^{4}$	Total	Bpcs
DPC	UE 1	1	0	0	0	55	0	5	97	73	359	5.61
	no modulo	2	0	0	10	110	3	52	117	12		
	UE 2	1	0	0	55	-	0	35	68	-	209	3.27
	modulo	2	0	10	110	-	6	88	12	-		
LP	UE 1	1	0	0	55	-	0	61	72	-	270	4.22
	no modulo	2	0	10	110	-	18	107	12	-		
	UE 2	1	0	0	55	-	0	34	67	-	204	3.19
	no modulo	2	0	10	110	-	5	86	12	-		

### 7.4. Simulation Results

**Figure 9:**
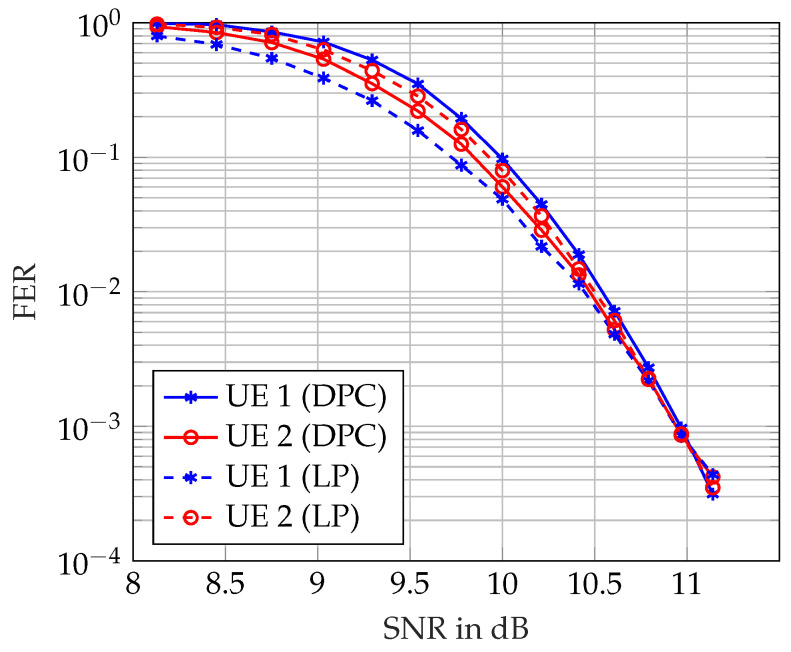
FER vs. $\mathrm{SNR}=P_{k}/4$ for DPC and LP with polar codes and the parameters of Table 1.

**Figure 10:**
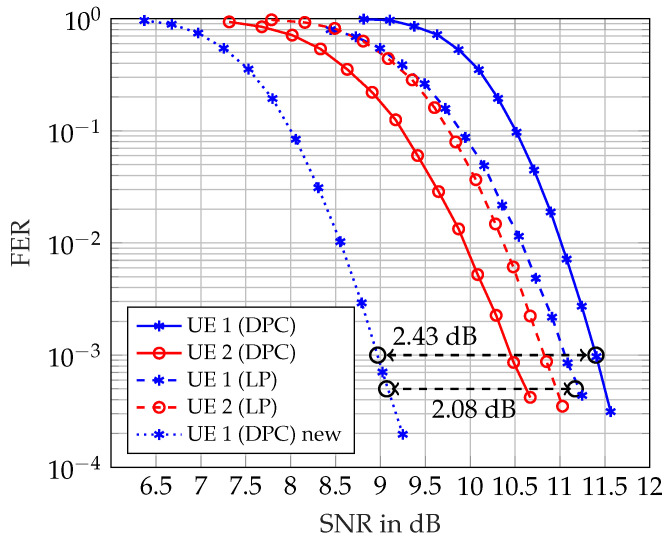
FER vs. $\mathrm{SNR}=P_{k}/2$ for DPC and LP with polar codes. The four curves on the right are taken from Figure 9. The leftmost curve is for the UE 1 (LP) code with $R_{1}=4.22$ bpcs.

The code design targeted $\mathrm{FER}=10^{-3}$ for $P_{X}=50$, or $P_{X}/4\approx 10.97$ dB. Figure 9 plots the FER for precoders that are optimized for each SNR. The DPC UE 1 code is ≈0.02 dB worse than the other codes at $\mathrm{FER}=10^{-3}$. Figure 10 replots the curves against the UE SNRs $P_{k}/2$ for k=1,2. The curves shift because the optimized UE powers $P_{1}$, $P_{2}$ are not exactly $P_{X}/2=25$:

$$\begin{array}{c} \begin{array}{ccc} \mathrm{DPC}: & P_{1}=27.65\;\left(0.44\;\mathrm{dB}\;\mathrm{shift}\right),\; & P_{2}=22.35\;\left(-0.49\;\mathrm{dB}\;\mathrm{shift}\right)\\ \mathrm{LP}: & P_{1}=25.65\;\left(0.11\;\mathrm{dB}\;\mathrm{shift}\right),\; & P_{2}=24.35\;\left(-0.11\;\mathrm{dB}\;\mathrm{shift}\right). \end{array} \end{array}$$

Next, for DPC and UE 1, we reduce the rate from 5.61 to 4.22 and design a code at $P_{1}=16.00$, or $P_{1}/2\approx 9.03$ dB, with optimized precoders. The result is the LP UE 1 code in Table 1 with the FER curve shown on the left in Figure 10. The UE 2 tone powers are kept constant here, so the UE 1 precoders do not change; only the waterfilling power allocation across the tones changes with $P_{1}$. The low-rate code achieves ≈2.43 dB gain over the original DPC UE 1 code, and the DPC structure achieves ≈2.08 dB gain over LP.

To better understand the ≈2.43 dB gain, consider the normal approximation for random coding on parallel AWGN channels [94] (Theorem 4). This approximation is

(92) $$\begin{array}{cc} \; & R\left(n,\mathrm{FER}\right)\approx \left[\sum_{t=1}^{T}C\left({\mathrm{SNR}}_{t}\right)\right]-\sqrt{\frac{\sum_{t=1}^{T}V\left({\mathrm{SNR}}_{t}\right)}{n}}\;Q^{-1}\left(\mathrm{FER}\right) \end{array}$$

(93) $$\begin{array}{cc} \; & C\left(x\right)=\frac{1}{2}\log_{2}\left(1+x\right),\;V\left(x\right)=\frac{x}{2}\frac{2+x}{{\left(1+x\right)}^{2}}\log_{2}{\left(e\right)}^{2} \end{array}$$

where *T* is the number of tones, *n* is the number of (real) symbols per tone, and ${\mathrm{SNR}}_{t}$ is the SNR of tone *t*. Our scenario has T=2 and n=128, and we wish to consider the UE 1 rates

(94) $$\begin{array}{c} R(n,\mathrm{FER})=5.61/2\approx 2.81,\;R(n,\mathrm{FER})=4.22/2\approx 2.11 \end{array}$$

in bits per real-alphabet channel symbol.

The tone SNRs are functions of $P_{1}$ because waterfilling changes the tone powers $P_{1,1}$ and $P_{1,2}$ as a function of $P_{1}$. We benchmark against the design power $P_{1}^{*}=16.00$ where waterfilling with the optimized precoders gives the tone singular values and powers

(95) $$\begin{array}{c} \sigma_{1,1}^{*}=2.81,\;\;P_{1,1}^{*}=8.32,\;\mathrm{and}\;\sigma_{1,2}^{*}=2.12,\;\;P_{1,2}^{*}=7.68. \end{array}$$

**Figure 11:**
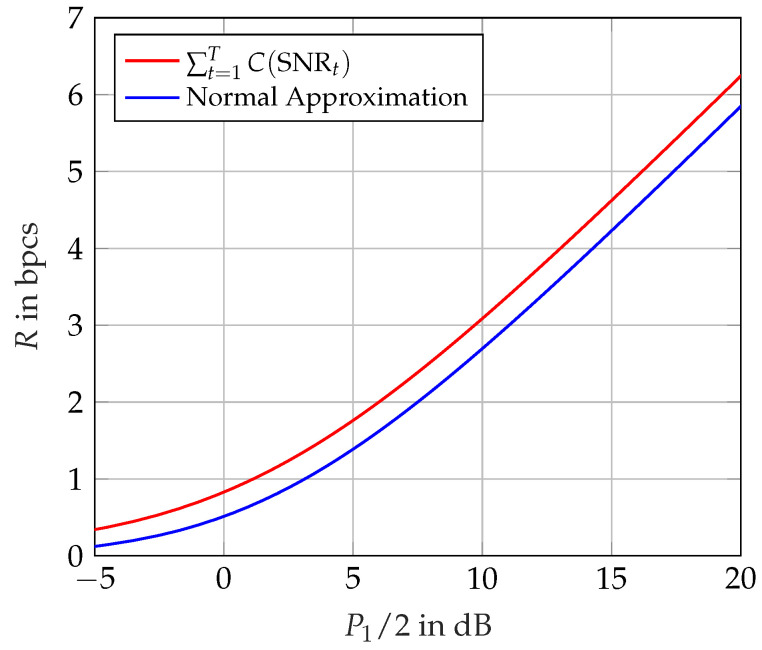
Rates vs. SNR. The blue curve is for T=2, n=128, and $\mathrm{FER}=10^{-3}$.

These singular values are close to the LP values in (77a), as expected, because the DPC and LP rates are similar despite UE 1 consuming less power. We now modify $P_{1}$, and recall that the UE 1 precoders do not change because the UE 2 tone powers are kept constant. The tone SNRs are thus

(96) $$\begin{array}{c} {\mathrm{SNR}}_{1}=\frac{{\left(\sigma_{1,1}^{*}\right)}^{2}}{P_{1,1}^{*}}\;P_{1,1}\left(P_{1}\right),\;{\mathrm{SNR}}_{2}=\frac{{\left(\sigma_{1,2}^{*}\right)}^{2}}{P_{1,2}^{*}}\;P_{1,2}\left(P_{1}\right) \end{array}$$

where we have written the tone powers as functions of $P_{1}$. Figure 11 plots the right-hand side of (92) and the rate $\sum_{t=1}^{T}C\left({\mathrm{SNR}}_{t}\right)$ against $P_{1}/2$. The rates (94) are achieved at $P_{1}/2\approx 10.37$ dB and $P_{1}/2\approx 7.91$ dB. The difference is ≈2.46 dB, which is close to the 2.43 dB shift of our multi-level polar codes.

### 7.5. Complexity Comparison

We compare the complexities. Determining the precoders $P_{k}$ of DPC and LP is a preprocessing step. Similarly, the SVD in (25) is a preprocessing step for computing the DPC receiver filters $F_{k}$ in (26), and one must similarly compute the LP receiver filters. Once these are determined, the linear operations for DPC and LP have the same complexity. Similarly, the DPC and LP polar decoders have the same complexities.

The polar encoding complexities, on the other hand, differ depending on whether shaping is used. With shaping, the encoding complexities are similar because DPC is essentially a shaping method; see (12). Without shaping, the LP polar encoder requires no decoding step. However, LP then loses the shaping gain, and we argue that the complexity advantage is minor.

- The DPC encoder may use SCL decoding with a small list size. We used SC decoding with a list size of 1, for which decoding can be further simplified [95,96].
- In our experiments, the UEs use SCL decoding with a list size of 8. Thus, the UE decoding complexity is at least 8 times the BS decoding complexity. The combined encoder–decoder complexity savings of LP without shaping can thus be approximated as being less then 1/9≈11%.
- The savings in encoding complexity occur at the BS. The relative savings are thus small, since a BS has many other computationally intensive tasks, including channel estimation, beamforming, resource allocation, scheduling, and so on.

We remark that one can implement the DPC structure in Figure 1 with a uniform shaping density q(x)=1/A for x∈[−A/2,A/2) rather than the truncated Gaussian (18). This approach is known as Tomlinson–Harashima (TH) precoding. At low SNR, the parameter $\alpha =P_{X}/\left(P_{X}+P_{Z}\right)$ is small, *X* is uniformly distributed over [−A/2,A/2), and the effective noise (15) is approximately uniformly distributed over [−A/2,A/2). Such noise is particularly harmful at low SNR, and the shaping gain is typically much larger than the maximal AWGN channel shaping gain of 1.53 dB; see [60] (Figure 3) and [61] (Section 3).

In summary, we believe that allowing shaping is fairest because it is necessary to approach capacity, it is less complex than decoding, and it comes for free with DPC. Also, the FER performance of the DPC structure in Figure 1 is similar with and without interference; see [62] (Figure 5), where the loss is ≈0.2 dB for n=256 real symbols and $\mathrm{FER}=10^{-3}$. The main issues for DPC are therefore not the complexities of precoding, decoding, or encoding. Instead, they are polar decoding latency, successive encoding latency (the dual of SIC decoding latency under NOMA or RSMA), performance under channel uncertainty, and compatibility with codes other than polar codes, e.g., LDPC codes.

## 8. Conclusions

We showed that scalar DPC based on modulo operations, probabilistic shaping, and dithering can approach any rate tuple in the capacity region of a complex-alphabet MIMO BC with CSCG noise and one message per receiver. The encoding and decoding complexities are similar to those of LP. Short polar code simulations demonstrate the rate gains achieved through DPC relative to LP. Future work may derive better bounds on achievable rates, e.g., by using informational divergence rather than bounds based on symmetric unimodal functions. One may also wish to better understand performance under channel uncertainty.

## Figures and Tables

**Figure A1:**
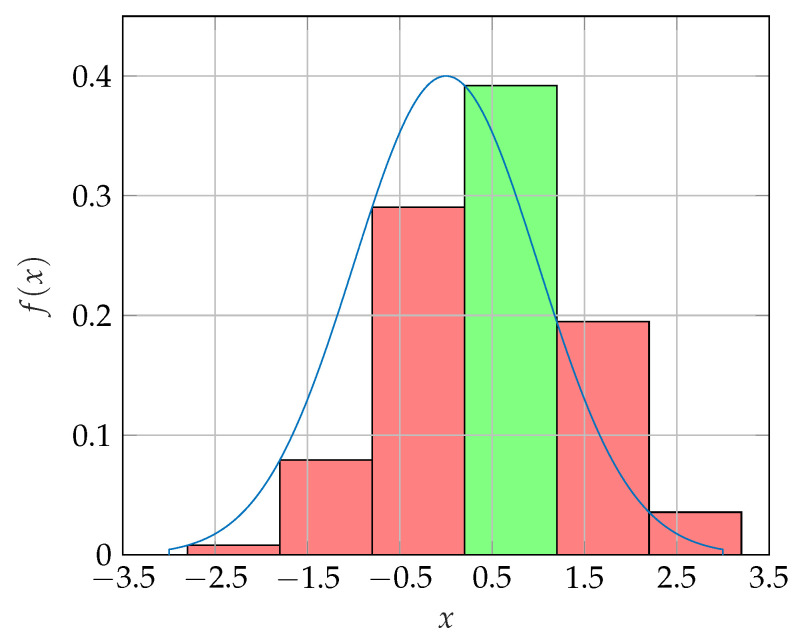
f(x) for A=M=6. The sampling points are shifted by *x* = 0.2 and are located at the top-left corner of each of the six bars.

**Figure A2:**
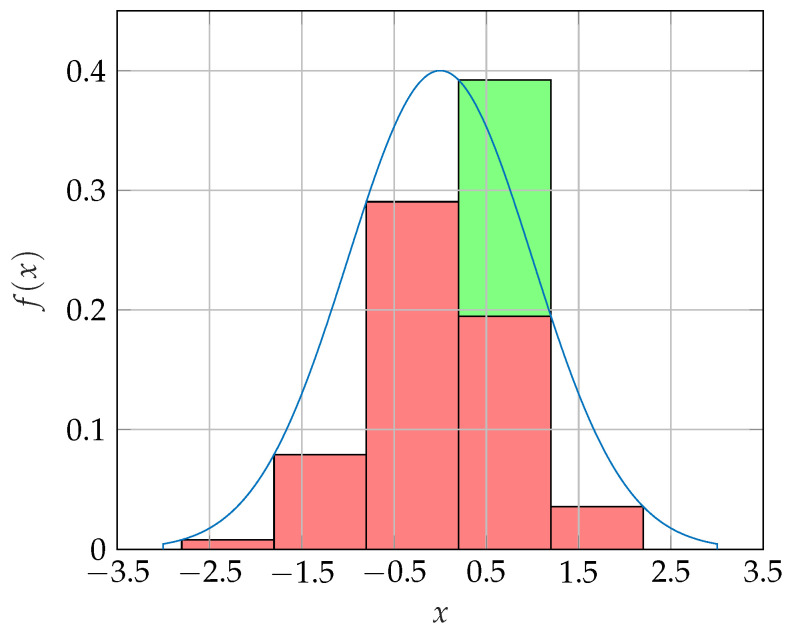
f(x) for A=M=6. The area of the red bars is less than the area of f(x), which is here 1.

**Figure A3:**
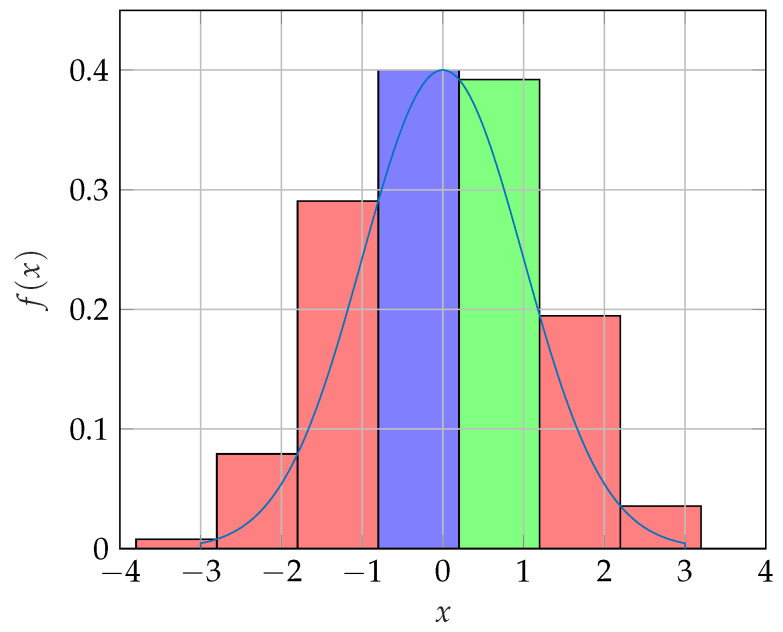
f(x) for A=M=6. The area of the red, green, and blue bars is greater than the area of f(x), which is here 1.

## Data Availability

The original contributions presented in this study are included in the article. Further inquiries can be directed to the corresponding author.

## Appendix A. Proof of Lemmas

### Appendix A.1. Proof of Lemma 1

If f(.) and g(.) are both symmetric then

(A1) $$\begin{array}{cc} f*g(-x) & =\int_{R}f\left(y\right)\;g\left(-x-y\right)\;dy\\ \; & \overset{\left(a\right)}{=}\int_{R}f\left(-y\right)\;g\left(x+y\right)\;dy\\ \; & \overset{\left(b\right)}{=}\int_{R}f\left(\tilde{y}\right)\;g\left(x-\tilde{y}\right)\;d\tilde{y}=f*g\left(x\right) \end{array}$$

where step (a) follows by symmetry and step (b) follows by substituting $\tilde{y}=-y$.

### Appendix A.2. Proof of Lemma 2

The lemma was proved in [97] (pp. 30–32) and [98] (Theorem 2.1); we here provide an alternative proof. Unimodality implies that p(.) can have a Dirac-delta component δ(.) at x=0 only, i.e., one may write

(A2) $$\begin{array}{c} p\left(.\right)=c_{p}\delta \left(.\right)+f\left(.\right) \end{array}$$

for a constant $c_{p}$ satisfying $0\leq c_{p}\leq 1$, and for a non-negative, symmetric, unimodal f(.) without δ(.) components. We may assume the derivative $f^{\prime }\left(.\right)$ exists almost everywhere [98] (Propositions 2.1). The *x* where $f^{\prime }\left(x\right)$ does not exist include “jumps” in f(.). For example, a negative “jump” at x≥0 from f(x)=a to f(x+ϵ)=b, where ϵ is a vanishing positive number and a>b≥0, becomes a (b−a)δ(.−x) component in $f^{\prime }\left(.\right)$.

As in (A2), consider $q\left(.\right)=c_{q}\delta \left(.\right)+g\left(.\right)$. We compute

(A3) $$\begin{array}{c} p*q\left(x\right)=c_{p}c_{q}\delta \left(x\right)+c_{p}g\left(x\right)+c_{q}f\left(x\right)+f*g\left(x\right) \end{array}$$

which is symmetric; see Lemma 1. The first three summands in (A3) are unimodal, so it remains to show that f∗g(.) is unimodal. Taking the derivative for x≥0, we have

(A4) $$\begin{array}{cc} {\left(f*g\right)}^{\prime }\left(x\right) & =\int_{-\infty }^{\infty }f\left(\tilde{y}\right)\;g^{\prime }\left(x-\tilde{y}\right)\;d\tilde{y}\\ \; & \overset{\left(a\right)}{=}\int_{0}^{\infty }\left[f(|x-y|)-f(x+y)\right]\;g^{\prime }\left(y\right)\;dy \end{array}$$

where step (a) follows by substituting $y=x-\tilde{y}$ and because f(.) and g(.) are symmetric. But the term in square brackets is non-negative because |x−y|≤x+y and f(.) is symmetric unimodal. We also have $g^{\prime }\left(y\right)\leq 0$ for y≥0 because g(.) is symmetric unimodal.

### Appendix A.3. Proof of Lemma 3

The sum in (7) is a Riemann sum, so we use the left and right rules of Riemann summation. Figure A1 shows a symmetric unimodal f(.) for κ=1 (here f(.) is a truncated Gaussian density). The sample points for x=0.2 are located at the top-left corner of each of the six bars. The area of each bar is one of the summands in (7); the area of the green bar is at most κf(0). Figure A2 shifts the red bars of positive sampling points to the left by κ, and we see that the area of the five red bars is less than the integral in (7). This can be done for any *x* with 0≤x<κ/2, so the sum in (7) is at most the integral plus κf(0).

Similarly, Figure A3 shifts the red bars of negative sampling points to the left by κ so they lie above f(x). If we add one (blue) bar of area κf(0), then the sum of the areas of the seven bars is greater than the integral in (7). This proves (7). To prove (8), we perform similar steps with a countable number of bars and appropriate left and right shifts. Note that we may restrict attention 0≤x<κ/2 by symmetry.

## Appendix B. Independence of the Channel Inputs ${\tilde{X}}_{k,i}$

Let ${\underline{\tilde{X}}}_{k,i}^{c}$ be the vector of all ${\tilde{X}}_{l,m}$ except ${\tilde{X}}_{k,i}$. Note from (24) that ${\tilde{S}}_{k,i}$ is a function of ${\underline{\tilde{X}}}_{k,i}^{c}$. Moreover, the chain ${\underline{\tilde{X}}}_{k,i}^{c}-{\tilde{S}}_{k,i}^{\prime }-U_{k,i}$ is Markov because $U_{k,i}$ is chosen using $S_{k,i}^{\prime }$ as shown in (12). Suppose the dithers $D_{k,i}$ are mutually independent, and consider the identities

(A5) $$\begin{array}{cc} p({\tilde{x}}_{k,i}|{\underline{\tilde{x}}}_{k,i}^{c})\; & =\int_{-A_{k,i}/2}^{A_{k,i}/2}p\left(d_{k,i}\right)\;p\left({\tilde{x}}_{k,i}|{\tilde{s}}_{k,i},d_{k,i}\right)\;dd_{k,i}\\ \; & \overset{\left(a\right)}{=}\sum_{u\in U_{k,i}}\frac{1}{A_{k,i}}\;\underset{\overset{\left(b\right)}{=}\kappa_{k,i}q\left({\tilde{x}}_{k,i}\right)/d\left({\tilde{x}}_{k,i}\right)}{\underset{︸}{P_{U_{k,i}|S_{k,i}^{\prime }}\left(u\left|(u-{\tilde{x}}_{k,i})\;\mathrm{mod}\;A\right.\right)}}\\ \; & \overset{\left(c\right)}{=}p\left({\tilde{x}}_{k,i}\right) \end{array}$$

where step (a) follows because ${\tilde{X}}_{k,i}$ is a discrete RV given $S_{k,i}^{\prime }=s_{k,i}^{\prime }$ (see (13)), step (b) follows by (12), (16) and (17) and step (c) follows because $\kappa_{k,i}=A_{k,i}/M_{k,i}$ and $p\left({\tilde{x}}_{k,i}\right)=q\left({\tilde{x}}_{k,i}\right)/d\left({\tilde{x}}_{k,i}\right)$ (see (16)). Thus, the channel inputs ${\tilde{X}}_{k,i}$ in (29) are mutually independent.

## Appendix C. Entropy Bounds

To prove (46a), let $I_{1}=\left\{x:q\left(x\right)<1\right\}$ and $I_{2}=\left\{x:q\left(x\right)\geq 1\right\}$. Observe that the length of $I_{2}$ is at most one since q(.) is a density. We bound

(A6) $$\begin{array}{cc} h(X) & \geq \int_{R}-p\left(x\right)\log\frac{q(x)}{d_{\min}}\;dx\\ \; & =\log d_{\min}-\int_{I_{1}}p\left(x\right)\log q\left(x\right)\;dx-\int_{I_{2}}p\left(x\right)\log q\left(x\right)\;dx\\ \; & \geq \log d_{\min}-\int_{I_{1}}\frac{q(x)}{d_{\max}}\log q\left(x\right)\;dx-\int_{I_{2}}\frac{q(x)}{d_{\min}}\log q\left(x\right)\;dx\\ \; & \;\pm \int_{I_{2}}\frac{q(x)}{d_{\max}}\log q\left(x\right)\;dx\\ \; & =\log d_{\min}+\frac{h_{q}\left(x\right)}{d_{\max}}-\left(\frac{1}{d_{\min}}-\frac{1}{d_{\max}}\right)\int_{I_{2}}q\left(x\right)\log q\left(x\right)\;dx\\ \; & \geq \log d_{\min}+\frac{h_{q}\left(x\right)}{d_{\max}}-\left(\frac{1}{d_{\min}}-\frac{1}{d_{\max}}\right){\left[q(0)\log q(0)\right]}^{+} \end{array}$$

where the final step follows because 1≤q(x)≤q(0) for $I_{2}\neq \emptyset$ and $x\in I_{2}$, and because $\int_{I_{2}}dx\leq 1$. Similar steps prove (46b) and (56).

## Appendix D. MAC Beamformers

The best MAC receiver beamformers are LMMSE filters. Let $K_{Y}$ be the covariance matrix of $\underline{Y}$. The LMMSE operation is the conditional expectation $E\left[X_{k}|\underline{Y}\right]={\bar{P}}_{k}^{†}\;\underline{Y}$ with the LMMSE filters

(A7) $$\begin{array}{cc} {\bar{P}}_{k}^{†} & =E\left[X_{k}\;{\underline{Y}}^{†}\right]\;K_{\underline{Y}}^{-1}. \end{array}$$

For example, for the channels (69), if the receiver decodes UE 1’s signal first, we have

(A8) $$\begin{array}{cc} {\bar{P}}_{1}^{†} & =\left[P_{1}h_{1}\;0\right]{\left[\begin{array}{cc} 1+P_{1}h_{1}^{2}+P_{2}h_{2}^{2} & P_{2}h_{2}^{2}\\ P_{2}h_{2}^{2} & 1+P_{2}h_{2}^{2} \end{array}\right]}^{-1}\\ \; & \propto \left[\begin{array}{cc} 1+P_{2}h_{2}^{2} & -P_{2}h_{2}^{2} \end{array}\right]. \end{array}$$

Similarly, decoding UE 2’s message first, we have

(A9) $$\begin{array}{cc} {\bar{P}}_{2}^{†} & =h_{2}P_{2}\;\left[1\;1\right]{\left[\begin{array}{cc} 1+P_{1}h_{1}^{2}+P_{2}h_{2}^{2} & P_{2}h_{2}^{2}\\ P_{2}h_{2}^{2} & 1+P_{2}h_{2}^{2} \end{array}\right]}^{-1}\\ \; & \propto \left[\begin{array}{cc} 1 & 1+P_{1}h_{1}^{2} \end{array}\right]. \end{array}$$

Observe that ${\bar{P}}_{1}$ is matched to $H_{1}$ for small $P_{2}$, and is orthogonal to $H_{2}$ for large $P_{2}$. Moreover, the LMMSE filter for transmitter 2, after removing the interference of transmitter 1, is matched to $H_{2}$. Similarly, ${\bar{P}}_{2}$ is matched to $H_{2}$ for small $P_{2}$, and is orthogonal to $H_{1}$ for large $P_{2}$. The LMMSE filter for transmitter 1, after removing the interference of transmitter 2, is matched to $H_{1}$.

## Appendix E. Dual MAC for LP

Consider the dual MAC, beamforming, and scaling in (63)–(65). The new SINR equivalences are (cf. (66) and [44])

(A10a) $$\begin{array}{cc} \; & \frac{|H_{1}P_{1}{|}^{2}}{1+|H_{1}P_{2}{|}^{2}}=\frac{|P_{1}^{†}H_{1}^{†}{|}^{2}P_{1}/a_{1}^{2}}{{∥P_{1}∥}^{2}/a_{1}^{2}+|P_{1}^{†}H_{2}^{†}{|}^{2}P_{2}/a_{1}^{2}} \end{array}$$

(A10b) $$\begin{array}{cc} \; & \frac{|H_{2}P_{2}{|}^{2}}{1+|H_{2}P_{1}{|}^{2}}=\frac{|P_{2}^{†}H_{2}^{†}{|}^{2}P_{2}/a_{2}^{2}}{{∥P_{2}∥}^{2}/a_{2}^{2}+|P_{2}^{†}H_{1}^{†}{|}^{2}P_{1}/a_{2}^{2}}. \end{array}$$

These equations simplify to

(A11a) $$\begin{array}{cc} \; & \frac{1}{1+|H_{1}P_{2}{|}^{2}}=\frac{P_{1}}{{∥P_{1}∥}^{2}+|H_{2}P_{1}{|}^{2}P_{2}} \end{array}$$

(A11b) $$\begin{array}{cc} \; & \frac{1}{1+|H_{2}P_{1}{|}^{2}}=\frac{P_{2}}{{∥P_{2}∥}^{2}+|H_{1}P_{2}{|}^{2}P_{1}}. \end{array}$$

Rearranging, we obtain

(A12) $$\left[\begin{array}{c} ∥P_{1}{∥}^{2}\\ ∥P_{2}{∥}^{2} \end{array}\right]=\underset{A}{\underset{︸}{\left[\begin{array}{cc} 1+|H_{1}P_{2}{|}^{2} & -|H_{2}P_{1}{|}^{2}\\ -|H_{1}P_{2}{|}^{2} & 1+|H_{2}P_{1}{|}^{2} \end{array}\right]}}\left[\begin{array}{c} P_{1}\\ P_{2} \end{array}\right]$$

and directly see the power equivalence (cf. (68))

(A13) $$\begin{array}{c} P_{1}+P_{2}=∥P_{1}{∥}^{2}+{∥P_{2}∥}^{2}. \end{array}$$

The matrix A in (A12) has an inverse with positive entries. Non-negative ${∥P_{1}∥}^{2},{∥P_{2}∥}^{2}$ thus give non-negative $P_{1},P_{2}$. The reverse may be seen by writing $P_{k}=∥P_{k}∥\;{\underline{u}}_{k}$ where $∥{\underline{u}}_{k}∥=1$ and inserting into (A11). We obtain

(A14a) $$\begin{array}{cc} {∥P_{1}∥}^{2}\left(1+a_{21}P_{2}\right)\; & =P_{1}\left(1+a_{12}{∥P_{2}∥}^{2}\right) \end{array}$$

(A14b) $$\begin{array}{cc} {∥P_{2}∥}^{2}\left(1+a_{12}P_{1}\right)\; & =P_{2}\left(1+a_{21}{∥P_{1}∥}^{2}\right). \end{array}$$

where $a_{kl}={\left|H_{k}\;{\underline{u}}_{l}\right|}^{2}$. Note that swapping $\left(P_{1},P_{2}\right)$ with $\left(∥P_{2}{∥}^{2},∥P_{1}{∥}^{2}\right)$ does not change the equations. Thus, non-negative $P_{1},P_{2}$ give non-negative ${∥P_{1}∥}^{2},{∥P_{2}∥}^{2}$; see (A12).

## Appendix F. Information Rates of LP with Two Modes

Consider LP with K=2 UEs and two transmission modes that are given fractions α and 1−α of the time/frequency resources. One may optimize over α, and the covariance matrices $K_{k,t}$ of each mode t=1,2. The following problem characterizes an achievable weighted sum-rate:

(A15) $$\begin{array}{cc} \; & \max\;w_{1}\left(\alpha R_{1,1}+\left(1-\alpha \right)R_{1,2}\right)+w_{2}\left(\alpha R_{2,1}+\left(1-\alpha \right)R_{2,2}\right)\\ \; & s.t.\;\alpha \mathrm{Tr}\left(K_{1,1}+K_{2,1}\right)+\left(1-\alpha \right)\mathrm{Tr}\left(K_{1,2}+K_{2,2}\right)\leq P_{X} \end{array}$$

where

(A16) $$\begin{array}{c} R_{k,t}=\log\left|I+H_{k}K_{k,t}H_{k}^{†}{\left(Q_{k}+H_{k}K_{3-k,t}H_{k}^{†}\right)}^{-1}\right|. \end{array}$$

For example, for the channels (69) with $n_{k}=1$, one should optimize α and the angles and powers of four precoding vectors (two precoding vectors for each UE).

## Appendix G. Rate Splitting

Consider $2^{K}-1$ independent vectors $X_{S}$ with ∅⊊S⊆{1,…,K}. The BS transmits

(A17) $$\begin{array}{c} X=\sum_{S}X_{S}. \end{array}$$

The idea is to split each message *k* into up to $2^{K-1}$ different messages, each carried by exactly one of the $X_{S}$ with k∈S. UE *k* decodes these $X_{S}$, either using joint decoding or SIC. Clearly, the number of parameters and bounds grows rapidly with *K*. For K=2, we have three messages and (70) is replaced with four decoding bounds:

(A18a) $$\begin{array}{cc} R_{\left\{1,2\right\}} & \leq \log\det\left(I+H_{k}K_{\left\{1,2\right\}}H_{k}^{†}{\left(Q_{k}+\sum_{l=1,2}H_{k}K_{\left\{l\right\}}H_{k}^{†}\right)}^{-1}\right) \end{array}$$

(A18b) $$\begin{array}{cc} R_{\left\{k\right\}} & \leq \log\det\left(I+H_{k}K_{\left\{k\right\}}H_{k}^{†}{\left(Q_{k}+\sum_{l\neq k}H_{k}K_{\left\{l\right\}}H_{k}^{†}\right)}^{-1}\right) \end{array}$$

for both k=1,2. We also have three rate-splitting bounds

(A19a) $$\begin{array}{cc} R_{1} & \leq R_{\left\{1\right\}}+R_{\left\{1,2\right\}} \end{array}$$

(A19b) $$\begin{array}{cc} R_{2} & \leq R_{\left\{2\right\}}+R_{\left\{1,2\right\}} \end{array}$$

(A19c) $$\begin{array}{cc} R_{1}+R_{2} & \leq R_{\left\{1\right\}}+R_{\left\{2\right\}}+R_{\left\{1,2\right\}}. \end{array}$$

For example, setting $K_{\left\{1,2\right\}}=0$, we have $R_{\left\{1,2\right\}}=0$ and obtain the two LP bounds (70). A more interesting case is $K_{\left\{1\right\}}=0$ so that $R_{\left\{1\right\}}=0$ and one chooses

(A20) $$\begin{array}{c} \left(R_{1},R_{2}\right)=\left(R_{\left\{1,2\right\}},R_{\left\{2\right\}}\right). \end{array}$$

In this case, UE 2 recovers both messages. As for LP, time- or frequency-sharing across multiple transmission modes can increase LP rates; see [15].
